# Asymptomatic herpes simplex virus brain infection elicits cellular senescence phenotypes in the central nervous system of mice suffering multiple sclerosis-like disease

**DOI:** 10.1038/s42003-024-06486-x

**Published:** 2024-07-04

**Authors:** Luisa F. Duarte, Verónica Villalobos, Mónica A. Farías, Ma. Andreina Rangel-Ramírez, Enrique González-Madrid, Areli J. Navarro, Javier Carbone-Schellman, Angélica Domínguez, Alejandra Alvarez, Claudia A. Riedel, Susan M. Bueno, Alexis M. Kalergis, Mónica Cáceres, Pablo A. González

**Affiliations:** 1 Millennium Institute on Immunology and Immunotherapy, Santiago, Chile; 2 Departamento de Ciencias Biológicas, Facultad de Ciencias de La Vida, Universidad Andrés Bello, Santiago, Chile; 3 Program of Cellular and Molecular Biology, Institute of Biomedical Sciences (ICBM), Faculty of Medicine, Universidad de Chile, Millennium Nucleus of Ion Channel-Associated Diseases (MiNICAD), Santiago, Chile; 4 Facultad de Ciencias Biológicas, Pontificia Universidad Católica de Chile, Santiago, Chile; 5 Departamento de Salud Pública, Facultad de Medicina, Pontificia Universidad Católica de Chile, Santiago, Chile; 6 Departamento de Endocrinología, Facultad de Medicina, Pontificia Universidad Católica de Chile, Santiago, Chile; 7 Present Address: Centro de Medicina Regenerativa, Facultad de Medicina, Clínica Alemana-Universidad del Desarrollo, Santiago, Chile

**Keywords:** Neural ageing, Autoimmunity, Virology, Chronic inflammation, Multiple sclerosis

## Abstract

Experimental autoimmune encephalomyelitis (EAE) is a demyelinating disease affecting the central nervous system (CNS) in animals that parallels several clinical and molecular traits of multiple sclerosis in humans. Herpes simplex virus type 1 (HSV-1) infection mainly causes cold sores and eye diseases, yet eventually, it can also reach the CNS, leading to acute encephalitis. Notably, a significant proportion of healthy individuals are likely to have asymptomatic HSV-1 brain infection with chronic brain inflammation due to persistent latent infection in neurons. Because cellular senescence is suggested as a potential factor contributing to the development of various neurodegenerative disorders, including multiple sclerosis, and viral infections may induce a premature senescence state in the CNS, potentially increasing susceptibility to such disorders, here we examine the presence of senescence-related markers in the brains and spinal cords of mice with asymptomatic HSV-1 brain infection, EAE, and both conditions. Across all scenarios, we find a significant increases of senescence biomarkers in the CNS with some differences depending on the analyzed group. Notably, some senescence biomarkers are exclusively observed in mice with the combined conditions. These results indicate that asymptomatic HSV-1 brain infection and EAE associate with a significant expression of senescence biomarkers in the CNS.

## Abstract (web summary)

Identification of senescence-related markers in the brain and spinal cord of mice with asymptomatic HSV-1 brain infection and animals undergoing experimental autoimmune encephalomyelitis which parallels multiple sclerosis in humans.

## Introduction

Multiple sclerosis is an autoimmune neurodegenerative disorder that affects the myelin sheath that covers nerve cells in the central nervous system (CNS) overall, leading to demyelination and axon degradation, producing physical, sensory, and cognitive disability problems^1,2^. At present, multiple sclerosis displays an increasing incidence and prevalence in the population and it is the primary cause of non-traumatic neurological dysfunction in young adults^3^. Its etiology is unknown, yet viral infections have been reported as potential triggers that could contribute to its onset, evolution, and severity of disease symptoms^4,5^. Thus, studying the effects of viral infections on multiple sclerosis may help identify molecular determinants contributing to the onset and development of this disease and help identify new strategies to prevent or treat multiple sclerosis.

Herpes simplex virus type 1 (HSV-1) is a neurotropic virus that is highly prevalent in the human population and causes lifelong infections, as it can remain latent in neurons with the possibility of periodic reactivations^6^. Infection with this virus may produce a broad spectrum of clinical manifestations, ranging from mild symptoms, such as oral and facial lesions, to severe disease of the eye and the central nervous system (e.g., encephalitis)^7,8^. 80–90% of individuals infected with this virus are asymptomatic, and importantly, ~35% of them could have asymptomatic brain infections with HSV-1, which has been associated with severe neuroimmune responses, chronic neuroinflammation and neurodegenerative diseases^9,10^. Previously, we reported that asymptomatic HSV-1 brain infection in mice after intranasal inoculation with this virus worsens a mild version of experimental autoimmune encephalomyelitis (EAE), a model used to study multiple sclerosis (Supplementary Fig. 1)^11^. We observed that HSV-1-infected animals with EAE displayed increased immune cell infiltration in the brain and spinal cord and significantly higher levels of interleukin (IL)-6, tumor necrosis factor-alpha (TNF-α), and IL-1β mRNA in these tissues (Supplementary Fig. 1)^11^. Importantly, there is increasingly compelling evidence that suggests that such an inflammatory environment is related to a senescence-associated secretory phenotype (SASP) in the CNS, which could be permissive for autoimmunity^12–15^.

Cellular senescence is a phenotype characterized by an irreversible cell cycle arrest with no proliferation and occurs due to different stimuli, including viral infections^13^. While there is no single molecular marker for defining cellular senescence, a hallmark of senescent cells is the acquisition of a complex proinflammatory secretory profile, named SASP, which consists mainly of the secretion of proinflammatory cytokines and chemokines, such as IL-6, CXC chemokine ligand 2 (CXCL-2), monocyte chemoattractant protein 1 (MCP-1/CCL2), matrix metalloproteinases (MMPs), serine proteases inhibitors (SERPINs), tissue inhibitors of metalloproteinases (TIMPs), and growth factors^16^. In addition, senescent cells can also display decreased expression of laminin B1 (Lmnb1) and increased expression of the cyclin-dependent kinase (CDK) inhibitors CDKN1a (p21^*Cip*1^) and CDKN2a (p16^*Ink*4*a*^), cellular components that are regulated by the p53 and retinoblastoma (pRB) proteins, respectively^14^. Moreover, senescent cells may display increased positive staining for senescence-associated-β-galactosidase (SA-β-Gal) in lysosomes, along with the formation of senescence-associated heterochromatin foci (SAHF), and DNA damage foci containing histone H2AX phosphorylated at serine 139 (γH2AX)^17^. Moreover, the relocalization of the high mobility group box 1 (HMGB1) protein from the nucleus to the cytoplasm acts as an alarmin of damage-associated molecular patterns (DAMPs) and has also been characterized as a molecular signature of senescent cells^18^.

Although cellular senescence plays beneficial roles in multiple biological processes, such as wound healing, tissue repair, and tumor suppression^19–21^, the accumulation of these cells in tissues has been associated with deleterious effects that predispose to age-related diseases and may be a root cause of neurodegenerative disorders^14,15^. Notably, senescence is not solely restricted to proliferating cells, as it has also been reported that a DNA damage response can induce a senescence-like phenotype in post-mitotic neurons in a p21-dependent way^22^. Moreover, increased post-mitotic senescence in aged human neurons has been reported as a pathological feature of Alzheimer’s disease in patients^23^. Given that there is accumulating evidence relating a senescence-like phenotype in neurons with aging-associated and neurodegenerative diseases, we hypothesized that asymptomatic brain infection with the neurotropic virus HSV-1 could induce a senescence-like state in neurons that may worsen in the context of EAE pathology^24–27^.

Therefore, we investigated the expression of molecular markers related to senescence within the brains and spinal cords of mice infected asymptomatically with HSV-1 through the intranasal route and then induced to develop EAE, alongside mice solely with HSV-1 asymptomatic infection, or mice with EAE alone. Moreover, we evaluated whether a senescence-like state was related to neurons under these conditions. Overall, we assessed if the senescence-related differences between the analyzed groups could relate to our earlier observation of increased demyelination and neuroinflammation in the CNS of mice with HSV-1 asymptomatic brain infection and EAE. Furthermore, we sought to determine molecular determinants that might contribute to the pathogenesis of these diseases and potentially serve as future therapeutic targets.

Our findings reveal that the conditions tested induce a significant increase in numerous senescence markers in the CNS upon EAE, irrespective of prior HSV-1 infection. Moreover, we observed that HSV-1-infected mice with EAE displayed a distinctive neuronal senescence-like state shedding light on molecular changes that are potentially triggered by asymptomatic HSV-1 brain infection over the CNS that may predispose to earlier and more severe CNS autoimmunity. These data support the notion that senotherapeutics may impact EAE and HSV-1-induced cellular senescence with potential applications for virus-induced neurodegenerative damage, which has yet to be determined experimentally.

## Results

### Increased expression of senescence molecular markers after asymptomatic HSV-1 brain infection and EAE

In a previous study, we reported an accelerated onset and increased severity of multiple sclerosis-like diseases after inducing a mild form of EAE in mice having prior asymptomatic HSV-1 brain infection^11^. In contrast to mock-EAE animals, which showed mild EAE symptoms followed by a remission stage post-peak, the animals with prior asymptomatic HSV-1 brain infection exhibited a markedly more severe and chronically progressive course of EAE symptoms that ultimately led to permanent paralysis (Supplementary Fig. 1a, reproduced with permission from ref. ^11^). This increase in clinical score, considered together with the duration of the disease further translated into a significantly higher area under the curve (AUC) value, which considers both the disease score and remission period (Supplementary Fig. 1b, reproduced with permission from ref. ^11^). Exacerbation of EAE in these mice was accompanied by increased immune cell infiltration and elevated mRNA levels of proinflammatory cytokines in both, brain, and spinal cord tissues (Supplementary Fig. 1c, d, reproduced with permission from ref. ^11^).

**Fig. 1:**
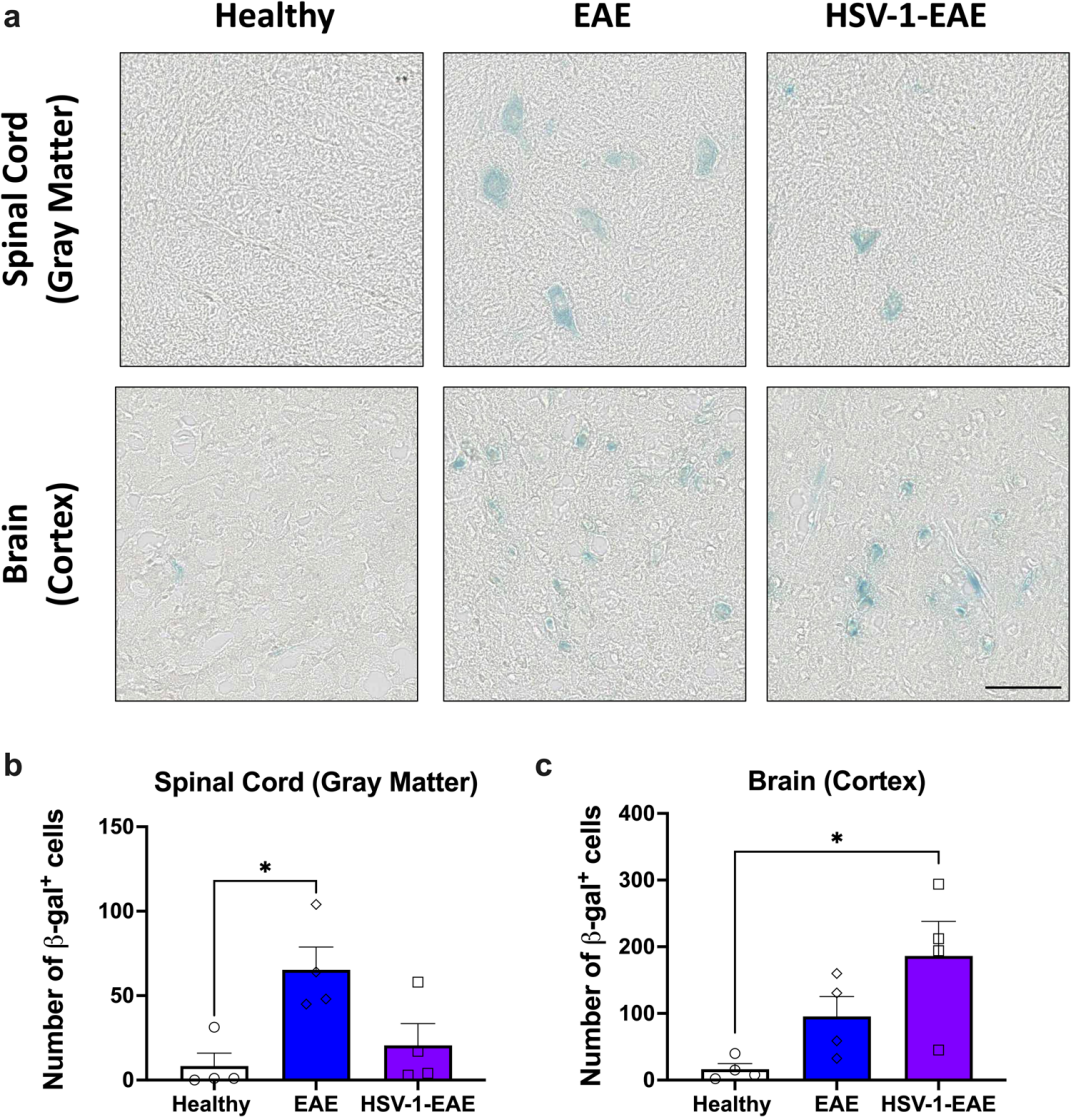
Brain and spinal cord tissues of mice with EAE show increased SA-β-Gal activity, regardless of previous HSV-1 infection. **a** Representative images for SA-β-Gal activity detection (blue staining) in the gray matter of spinal cord and brain cortex tissues of healthy mice and mice with EAE, with or without previous HSV-1 infection. Measurement of SA-β-Gal activity was carried out in tissues fourteen days after EAE induction. Images correspond to 20X magnifications and the scale bar to 100 μm. **b** Quantification of SA-β-Gal-positive cells in the gray matter of spinal cord tissue. **c** Quantification of SA-β-Gal-positive cells in the brain cortex tissue. White bars with circles represent data from healthy mice, blue bars with diamonds represent data from mice with EAE and purple bars with squares represent data from mice with EAE and HSV-1 infection. Values represent means ± SEM of four animals per group. Data were analyzed using the Kruskal–Wallis test followed by Dunn’s post-hoc test; *p < 0.05.

Given that neuroinflammation is a common feature in asymptomatic HSV-1 infection and EAE that may be associated with inflammatory SASP factors related to cellular senescence, we sought to investigate in this study the presence of a senescent phenotype in cells within brain and spinal cord tissues under these experimental settings. As an initial approach, we evaluated the activity of SA-β-Gal, which is an inducible lysosomal enzyme that exhibits augmented activity levels during senescence. Interestingly, we observed a substantial increase of SA-β-Gal activity in the brain cortex and the gray matter of spinal cord tissues of mice obtained 14 days after EAE induction, regardless of prior HSV-1 infection, compared to healthy control mice (Fig. 1a). Quantification of the number of cells exhibiting SA-β-Gal activity evidenced a significant increase in such positive cells in spinal cord tissue of mice with EAE, as compared to healthy controls (Fig. 1b). In brain tissues, a significant increase in the number of cells exhibiting SA-β-Gal activity could be observed for the HSV-1-EAE group, as compared to healthy mice (Fig. 1c).

**Fig. 2:**
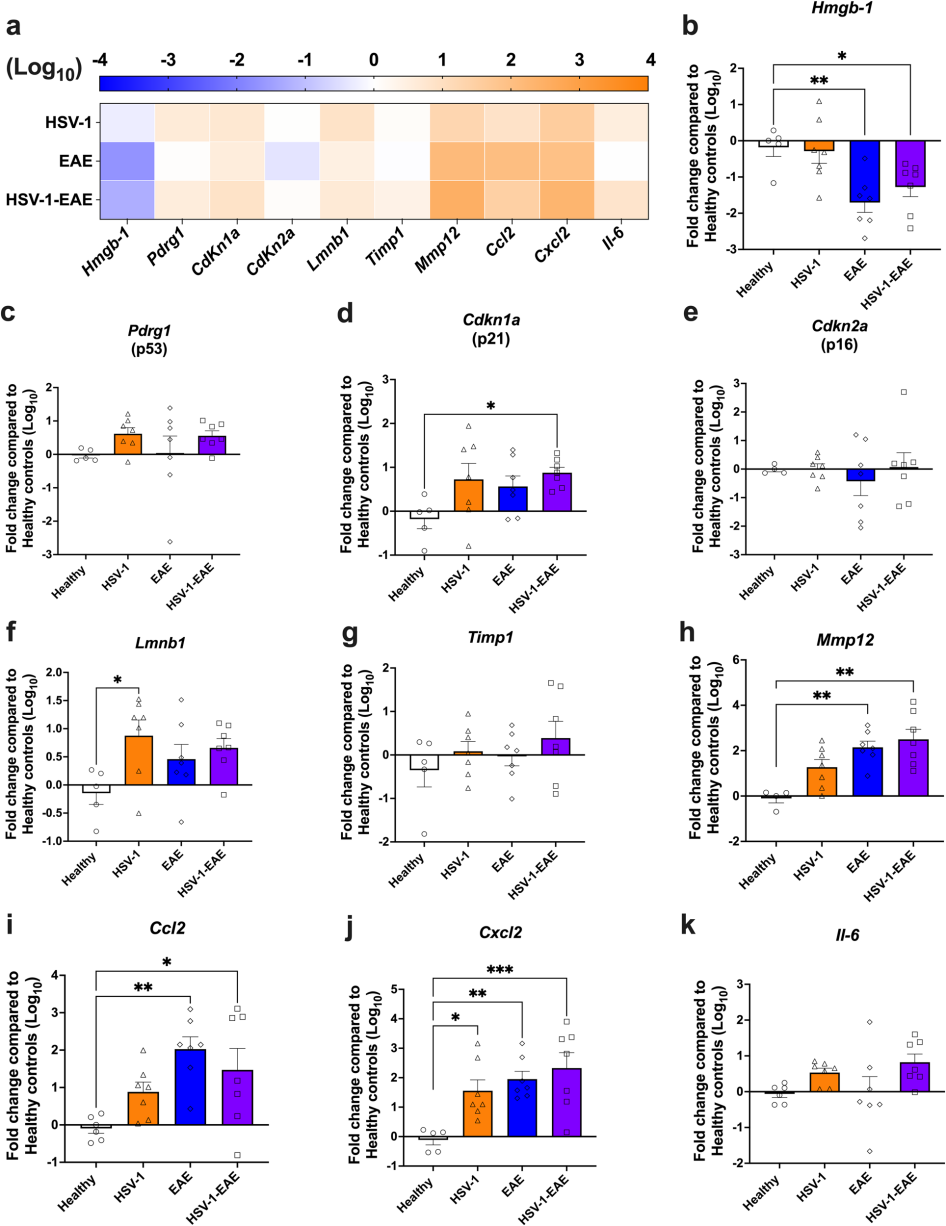
Asymptomatic HSV-1 brain infection and EAE, individually or combined, increase the mRNA levels of senescence-associated genes in the spinal cord. Mice were mock-treated (healthy group, white bars with circles) or asymptomatically infected with HSV-1 strain 17syn^+^ in the brain (HSV-1 group, orange bars with triangles). EAE was induced 4 weeks after mock treatment (EAE group, blue bars with diamonds), or infection (HSV-1-EAE group, purple bars with squares). Spinal cord homogenates were recovered 14 days after EAE induction, or 45–50 days after HSV-1 infection, or mock treatment alone. The expression of senescence-associated genes was evaluated at the mRNA level by RT-qPCR using the 2^−ΔΔCT^method with *β-actin* as a reference gene. **a** Heatmap comparing mRNA levels of senescence-associated genes in the spinal cord of HSV-1, EAE, and HSV-1-EAE groups. The orange color indicates upregulation while the blue color indicates downregulation. Darker colors indicate stronger effects. Relative mRNA expression of the gene products **b** *Hmgb-1*, **c** *Pdrg1*, **d** *Cdkn1a*, **e** *Cdkn2a*, **f** *Lmnb1*, **g** *Timp1*, **h** *Mmp12*, **i** *Ccl2*, **j** *Cxcl2*, and **k** *Il6* in the different mouse groups compared to healthy controls. Values represent means ± SEM of two independent experiments (*n* = 7 animals/group). Log-transformed data were analyzed using one-way ANOVA followed by Dunnett’s post-hoc test; ***p < 0.001, **p < 0.01, and *p < 0.05.

These findings prompted us to further characterize additional senescence markers that may be elicited in EAE, with or without previous HSV-1 asymptomatic brain infection. To further elucidate the impact of HSV-1 infection on the generation of a premature senescence state in the CNS, a group of mice that underwent asymptomatic HSV-1 brain infection without EAE was also included. For this, we performed RT-qPCR analyses of spinal cord and brain tissues recovered 14 days after EAE induction from mice with EAE alone and HSV-1 infection plus EAE, as well as 45 days after HSV-1 infection (HSV-1 group), evaluating the mRNA levels of a set of 10 genes identified as senescence hallmarks, namely *Lmnb1*, a collection of SASP factors (*Hmgb-1, Timp1, Mmp12, Ccl2, Cxcl2*, and *Il6*), the CDK inhibitors *Cdkn1a* (gene of the p21 protein) and its regulatory component *Pdrg1* (gene of the p53 protein), as well as *Cdkn2a* (gene of the p16 protein). As shown in the heatmap and graphs in Fig. 2a–k, a global trend towards increased mRNA levels associated with senescence was evident across spinal cord tissues in all the examined groups, except for the *Hmgb-1* gene, which was downregulated (Fig. 2b). Notably, the group with HSV-1-infected mice plus EAE (HSV-1-EAE) showed a statistically significant increase in the mRNA levels of the *Cdkn1a* gene when compared to the healthy control group (Fig. 2d). Moreover, a significant upregulation in mRNA levels of the *Mmp12* and *CCL2 genes* was observed in both groups with EAE, which was approximately 20-fold higher as compared to the healthy control group (Fig. 2h, i). Notably, all experimental groups showed a significant increase in the mRNA levels of the *Cxcl2* gene when compared to the healthy counterparts (Fig. 2j). Finally, an additional assessment was conducted 21 days after EAE induction (remission stage), as well as 52 days after HSV-1 infection for a subset of these genes (Supplementary Fig. 2a–f). Interestingly, we observed a significant increase in the mRNA levels of the *Hmgb-1* gene in the HSV-1 group without EAE (Supplementary Fig. 2b), and mRNA levels of the *Cdkn2a* gene in the EAE group as compared to healthy mice (Supplementary Fig. 2d). In addition, mRNA levels of the *Mmp12* gene remained elevated in both EAE groups when compared to the control group (Supplementary Fig. 2e).

Given the observed upregulation of mRNA levels of certain genes associated with senescence in the spinal cord of HSV-1-infected mice without EAE, we sought to investigate whether the virus reaches this organ following intranasal infection. Thus, we conducted qPCR assays to detect viral loads in the brain, spinal cord, and trigeminal ganglia of the infected animals at two different timepoints after intranasal HSV-1 infection. Surprisingly, we found the presence of the viral genome in the spinal cord tissues at 4 days post-infection (d.p.i), which was almost completely cleared by 30 d.p.i. A similar finding was observed in brain tissues. As expected, HSV-1 genetic material persisted throughout the analyzed timepoints in the trigeminal ganglia (Supplementary Fig. 3). The presence of HSV-1 genetic material in brains and spinal cords suggests the possibility of direct virus-elicited effects at these sites, as well as potential indirect alterations therein due to neuroinflammatory processes spreading from the infected tissues to cells in the nearby environment.

**Fig. 3:**
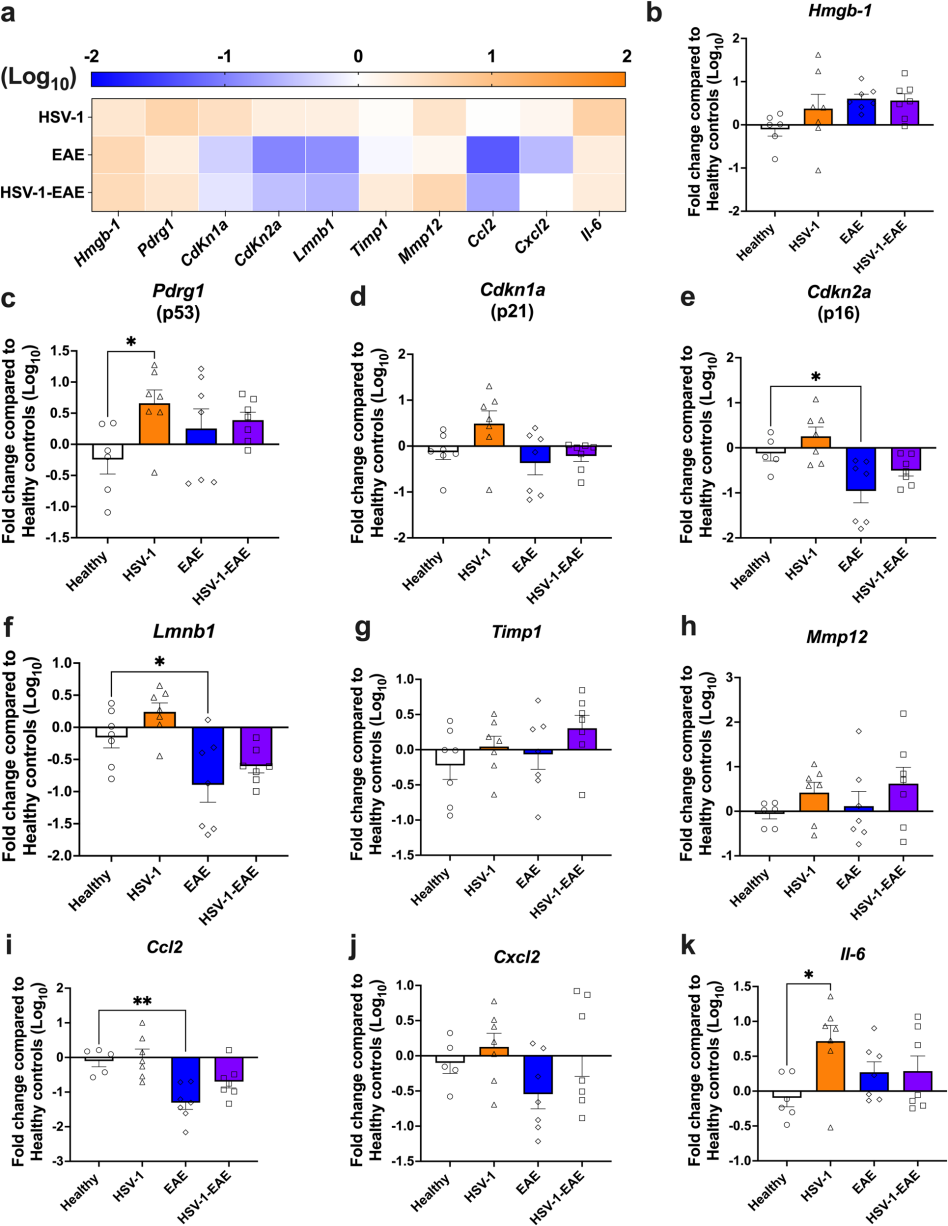
Asymptomatic HSV-1 brain infection and EAE, individually or combined, increase the mRNA levels of senescence-associated genes in the brain. Mice were mock-treated (healthy group, white bars with circles) or asymptomatically infected with HSV-1 in the brain (HSV-1 group, orange bars with triangles). EAE was induced four weeks after mock treatment (EAE group, blue bars with diamonds) or infection (HSV-1-EAE group, purple bars with squares). Brain homogenates were recovered fourteen days after EAE induction, 45–50 days after HSV-1 infection, or mock treatment alone. The expression of senescence-associated genes was evaluated at the mRNA level by RT-qPCR using the 2^−ΔΔCT^method with *β-actin* as a reference gene. **a** Heatmap comparing the mRNA levels of senescence-associated genes in the brain among HSV-1, EAE, and HSV-1-EAE groups. The orange color indicates upregulation while the blue color indicates downregulation. Darker colors indicate stronger effects. Relative mRNA expression of the gene products **b** *Hmgb-1*, **c** *Pdrg1*, **d** *Cdkn1a*, **e** *Cdkn2a*, **f** *Lmnb1*, **g** *Timp1*, **h** *Mmp12*, **i** *Ccl2*, **j** *Cxcl2*, and **k** *Il6* in the different mouse groups compared to the healthy controls. Values represent means ± SEM of two independent experiments (*n* = 7 animals/group). Log-transformed data were analyzed using one-way ANOVA followed by Dunnett’s post-hoc test; **p < 0.01 and *p < 0.05.

On the other hand, an increase in the mRNA levels of SASP-associated genes was less pronounced in brain tissues in the EAE groups, as evidenced in the heatmap and graphs shown in Fig. 3a–k. A significant increase in of *Pdrg1* mRNA levels was observed, but only in the HSV-1 group without EAE (Fig. 3c). Furthermore, the groups with EAE exhibited downregulated levels of *Lmnb1* mRNA, which is related to senescence. However, this difference was only statistically significant when comparing the EAE group without prior HSV-1 infection to the healthy control group (Fig. 3f). Animals previously infected with HSV-1 also showed a significant upregulation in mRNA levels of the *Il6* gene compared to healthy mice (Fig. 3k). Similar results were observed at the other timepoint assessed (Supplementary Fig. 2g–l), with animals in the HSV-1 infection-only group displaying a significant upregulation of mRNA levels associated to cell cycle arrest (*Cdkn1a* and *Cdkn2a)* and the gene of IL-6 *(Il6)*, as compared to the healthy controls (Supplementary Fig. 2i, j, l).

These results unveil a heterogeneous expression of senescence-related molecular markers triggered by asymptomatic HSV-1 brain infection and EAE, which varies depending on specific CNS tissues and timepoints evaluated. It is important to note that there was a distinctive senescence molecular pattern related to previous asymptomatic HSV-1 infection in the brains and spinal cords, which was characterized by the upregulation of mRNA levels of genes associated with cell cycle arrest (*Cdkn1a*, *Pdrg1*, *and Cdkn2a*), along with the SASP components (*Il6, Cxcl2*, *and Hmgb-1)*. Regarding EAE, a senescent phenotype was more evident in the spinal cord, which was characterized by increased mRNA levels of genes related to cell chemoattraction and MMPs.

### Asymptomatic HSV-1 brain infection, EAE, and their combined effects induce DNA damage markers in neurons within the CNS

**Fig. 4:**
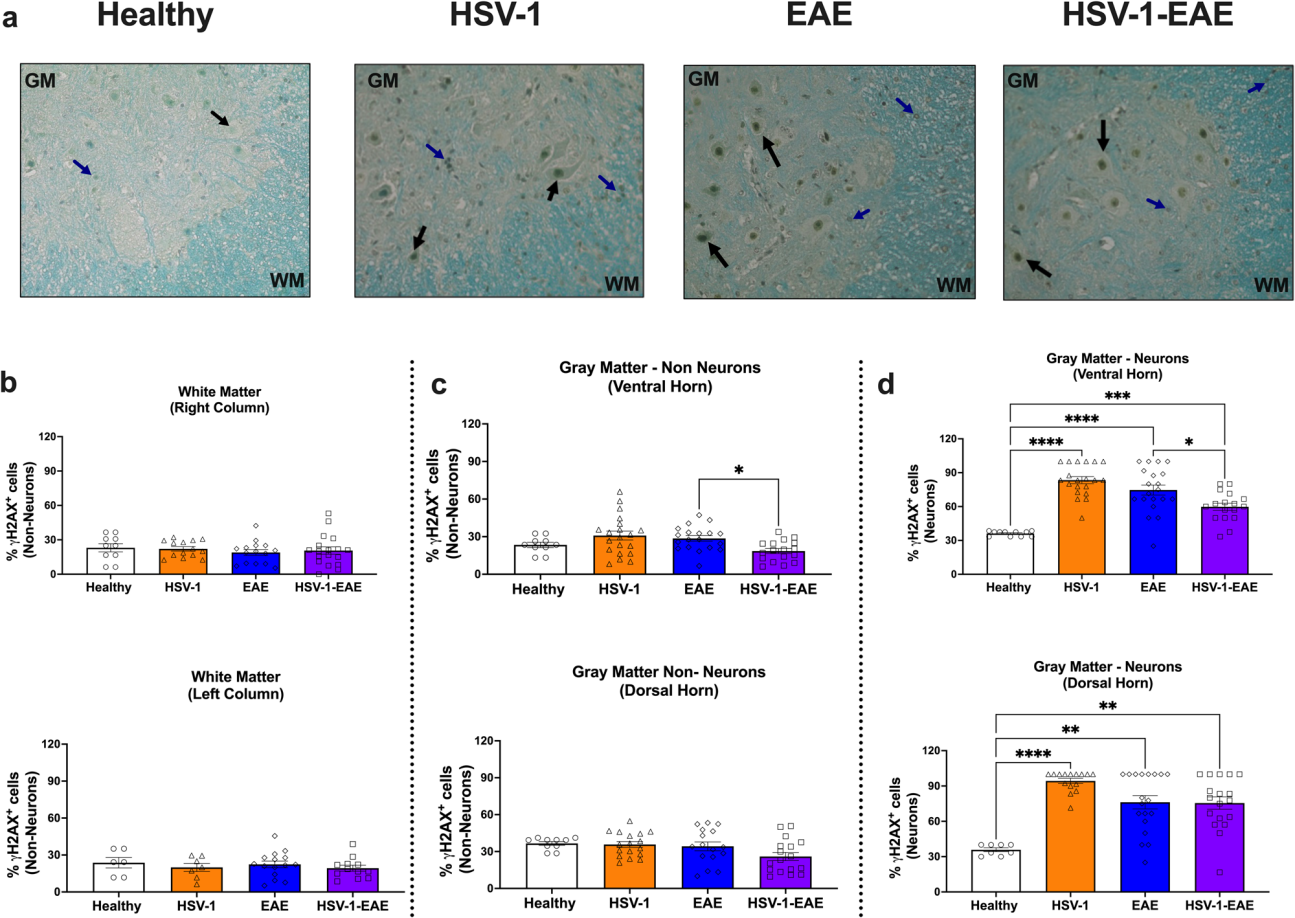
Asymptomatic HSV-1 brain infection and EAE, individually or combined, induce DNA damage-related foci in spinal cord neurons. Spinal cord tissue was harvested 14 days after EAE induction, 45–50 days after HSV-1 infection, or mock-treatment alone for detecting the phosphorylation of histone H2AX (γH2AX) by immunohistochemistry. **a** Representative images showing DNA damage-related γH2AX foci in neurons (black arrows) and non-neuron cells (blue arrows). GM: gray matter; WM: white matter. **b** Quantification of γH2AX foci in the nucleus of non-neuron cells in the white matter (analysis separated in right and left columns, upper and lower panels, respectively). **c** Quantification of γH2AX foci in the nucleus of non-neuron cells in the gray matter (analysis separated in ventral and dorsal horns, upper and lower panels, respectively). **d** Quantification of γH2AX foci in the nucleus of neuron cells in the gray matter (analysis separated in ventral and dorsal horns upper and lower panels, respectively). White bars with circles represent data from healthy mice, orange bars with triangles represent data from mice with HSV-1 infection, blue bars with diamonds represent data from mice with EAE and purple bars with squares represent data from mice with EAE and HSV-1 infection. Values represent means ± SEM of the percentage of γH2AX-positive cells of four mice per group. Data were analyzed using One-way ANOVA followed by Bonferroni’s post-hoc test for multiple comparisons with healthy control mice (*n* = 2); ****p < 0.001, **p < 0.01, *p < 0.05.

Given that asymptomatic HSV-1 brain infection and EAE elicit senescence-like signatures at the mRNA level in bulk brain and spinal cord samples, we sought to assess the presence of additional senescence-related markers at the protein level in these tissues. For this, we analyzed histone H2AX phosphorylated at serine 139 (γH2AX), a marker of DNA damage associated with senescence^28,29^, in spinal cord tissue to assess alterations linked to a specific cellular compartment by immunohistochemistry (Fig. 4a, representative images are shown). Based on the cellular distribution within the spinal cord, we separated the quantification of γH2AX-positive cells within the white and gray matter in different regions. The white matter, mostly comprised of cell nuclei of non-neuron cells, was further subdivided into spinal cord left and right columns. In contrast, the gray matter, consisting of neurons and other non-neuronal cells (distinguished morphologically), was divided into anterior and posterior horns. Interestingly, quantifications performed at 14 days after EAE induction (EAE and HSV-1-EAE groups), and 45 days after viral infection (HSV-1 group) did not reveal any significant differences in the percentage of non-neuron cells with nuclear γH2AX staining across all evaluated experimental groups when compared to the healthy control group in the white and gray matter (Fig. 4b, c, respectively).

Conversely, in the gray matter, there was a significant increase in the percentage of γH2AX-positive neurons in all experimental groups evaluated, as compared to healthy mice, both in the ventral and dorsal horns (Fig. 4d). Similar findings were observed at an additional timepoint carried out 21 days after EAE induction, in the remission stage, and 52 days after HSV-1 infection (Supplementary Fig. 4). Notably, at this timepoint, a significant increase in the percentage of non-neuron cells displaying nuclear foci associated with DNA damage was observed in the EAE and HSV-1-EAE groups, in the gray matter and white matter, respectively (Supplementary Fig. 4).

**Fig. 5:**
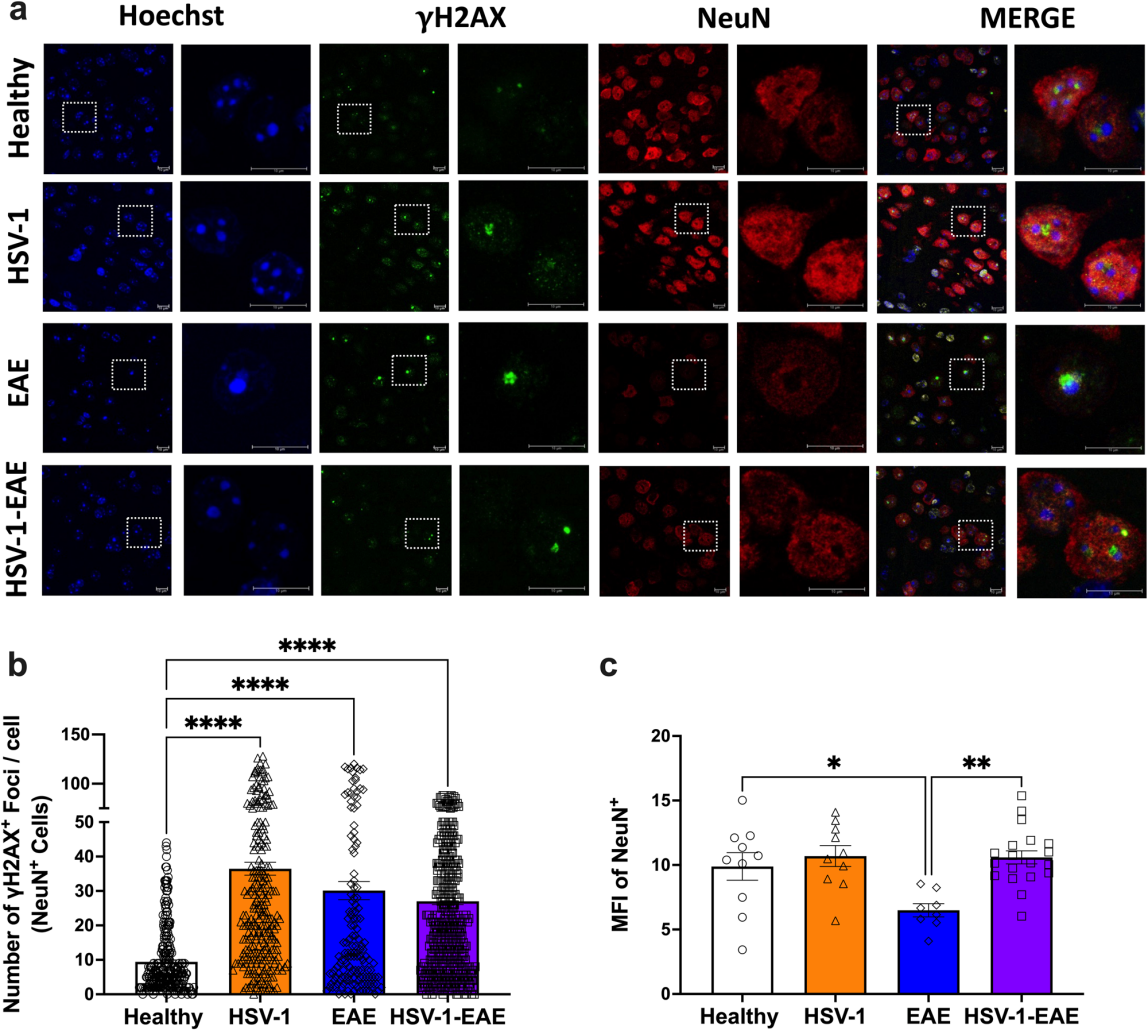
Asymptomatic HSV-1 brain infection and EAE, individually or combined, induce DNA damage-related foci in brain neurons. Brain tissues from four animals per group were harvested fourteen days after EAE induction or 45–50 days after asymptomatic HSV-1 brain infection or mock treatment alone for detecting the phosphorylation of histone H2AX (γH2AX) by immunofluorescence. **a** Representative images showing Hoechst nuclei staining (blue), γH2AX straining (green), NeuN staining (red), and image merges. Left: images for each fluorescence channel correspond to 100X magnifications and right: images are shown at a 5X optic zoom of the area outlined in squares with white dashed lines. Scale bars = 10 μm. **b** Quantification of γH2AX foci in the nucleus of NeuN-positive cells. Values represent means ± SEM of the measurements carried out in at least ten fields per sample. Data were analyzed using one-way ANOVA followed by Bonferroni’s post-hoc test; ****p < 0.0001. **c** Quantifi**c**ation of the mean fluorescence intensity (MFI) of the NeuN staining. Data were analyzed using Kruskal–Wallis followed by Dunn’s post-hoc test; **p < 0.01, *p < 0.05. White bars with circles represent data from healthy mice, orange bars with triangles represent data from mice with HSV-1 infection, blue bars with diamonds represent data from mice with EAE and purple bars with squares represent data from mice with EAE and HSV-1 infection.

Next, we carried out immunofluorescence analyses to determine whether γH2AX-positive staining, which is related to DNA damage was also triggered in neurons in the brain tissue at 14 days after EAE induction (EAE and HSV-1-EAE groups), and 45 days after viral infection (HSV-1 group). For this, we identified neuronal cell nuclei in the brain cortex using the neuronal nuclear marker NeuN and quantified γH2AX foci in a blinded manner regarding specimen identity (Fig. 5a). Microscopy analyses revealed that the number of γH2AX foci per NeuN-positive cells (Fig. 5b) was higher in mice previously infected with HSV-1, mice with EAE, and mice with concomitant HSV-1 infection and EAE, as compared to healthy animals. In addition, we performed immunofluorescence staining to determine DNA damage in another cell type, namely oligodendrocyte progenitor cells (OPCs) using the marker A2B5 and assessing senescence by detecting the p53-binding protein 1 (53BP1) as a early response to DNA double-strand breaks. In this case, we observed the limited formation of 53BP1 nuclear foci in these cells (Supplementary Fig. 5a). Furthermore, we quantified the number of foci associated with DNA damage in NeuN-negative cells. Interestingly, the overall count of foci observed in this case was lower than that observed in NeuN-positive cells (∼1 vs ∼35, respectively) (Supplementary Fig. 5b).

Notably, we detected a decrease in the mean fluorescence intensity (MFI) of the NeuN staining in mice with EAE at 14 days after EAE induction, suggesting neuronal loss, which was statistically significant when compared to healthy controls (Fig. 5c). This result is consistent with previous findings reported by other research groups studying EAE models and other cognitive impairments^18,30,31^. Surprisingly, we observed comparable MFI values for NeuN in the brains of mice with prior HSV-1 infection and EAE as compared to healthy mice. This observation could be related to the capacity of HSV-1 infection to modulate neuronal survival in the brain to favor its persistence and shedding throughout this tissue^6^.

In summary, these results suggest that neurons in HSV-1-infected mice, as well as animals with EAE undergo DNA damage, which could trigger a senescence state in the CNS as observed in these mice.

### Asymptomatic HSV-1 brain infection elicits neuronal senescence-like markers in mice with EAE characterized by HMGB-1 translocation to the cytoplasm and SAHF formation

**Fig. 6:**
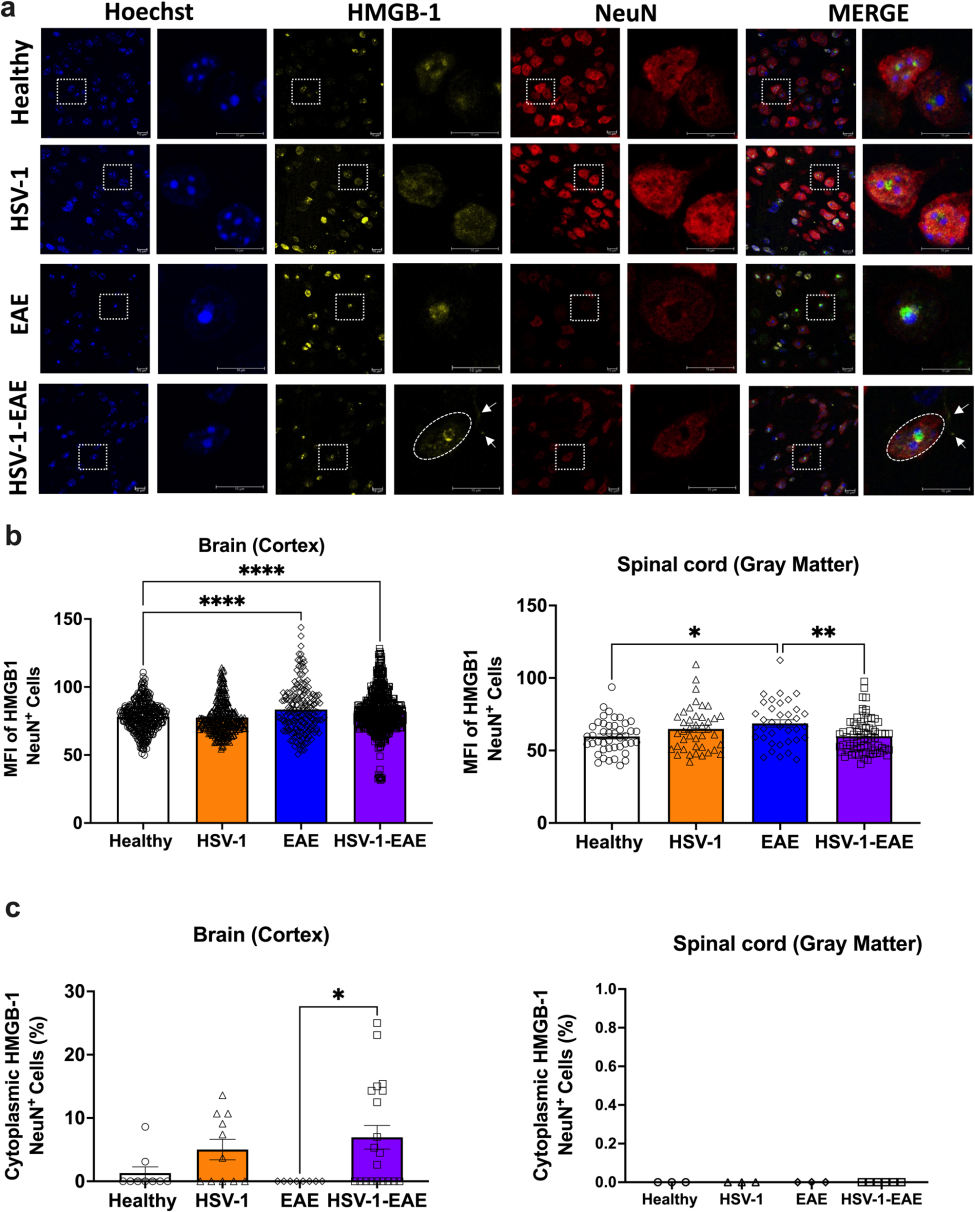
Asymptomatic HSV-1 brain infection and following EAE produce HMGB-1 release from the nucleus to the cytoplasm in brain neurons. Brain and spinal cord tissues from four animals per group were harvested 14 days after EAE induction, 45–50 days after HSV-1 infection, or mock-treatment alone for detecting the expression and release of HMGB-1 by immunofluorescence. **a** Representative images showing Hoechst nuclei staining (blue), HMGB-1 staining (yellow), NeuN staining (red), and image merges. Left: images in each fluorescence channel correspond to 100X magnifications, and right: images are shown at a 5X optic zoom of the area outlined in squares with white dashed lines. Scale bars = 10 μm. White arrows show the translocation of HMGB-1 from the nucleus to the cytoplasm. **b** Quantification of the mean fluorescence intensity (MFI) of HMGB-1 in the nucleus of NeuN^+^ cells in the brain cortex (left), and in the gray matter of the spinal cord (right). **c** Percentage of NeuN^+^ cells with HMBG-1 release from the nucleus toward cytoplasm in the brain cortex (left) and in the gray matter of the spinal cord (right). Values represent means ± SEM of the measurements carried out in at least ten fields in the brain tissues and three fields in the spinal cord tissues per sample. Data of MFI were analyzed using one-way ANOVA followed by Bonferroni’s post-test, and data of cytoplasmic HMGB-1 were analyzed using Kruskal–Wallis followed by Dunn’s post-hoc test; ****p < 0.0001, **p < 0.01, *p < 0.05. White bars with circles represent data from healthy mice, orange bars with triangles represent data from mice with HSV-1 infection, blue bars with diamonds represent data from mice with EAE and purple bars with squares represent data from mice with EAE and HSV-1 infection.

Another molecular hallmark in senescent cells is the release of HMGB-1 from the nucleus to the cytoplasm. HMGB-1 acts as an alarmin that induces tissue deterioration through senescence-associated inflammation, and elevated levels of this protein have been reported in patients with multiple sclerosis and Alzheimer’s disease^32,33^. Moreover, HMGB-1 has been shown to inhibit the differentiation of oligodendrocyte progenitor cells and restrict CNS remyelination^34^. Here, we performed immunofluorescence assays to assess the expression and location of HMGB-1 in neurons in brain and spinal cord tissues in the analyzed mouse groups (Fig. 6a), similar to other reports^35–37^. Microscopy analysis, which was carried out blindly, showed that the MFI of HMGB-1 appears to slightly increase in NeuN-positive cells within the brain tissue of mice with EAE, either with or without previous asymptomatic HSV-1 brain infection, as compared to control mice (Fig. 6b). Conversely, in the spinal cord tissue, a slight, but significant increase in MFI was observed for HMGB-1 only in neurons in the EAE group without infection (Fig. 6b). However, further studies will be needed to elucidate the biological relevance of these observed changes in HMGB-1.

Interestingly, animals previously infected with HSV-1 and undergoing EAE exhibited a significant increase in the percentage of NeuN-positive cells displaying translocation of HMGB-1 from the nucleus to the cytoplasm in the brain tissue (Fig. 6c). Here, we also evaluated the localization of HMGB-1 in activated microglia using the IBA-1 marker to identify these cells from others (Supplementary Fig. 6a) and quantified the percentage of NeuN-negative cells with HMGB-1 being released into the cytoplasm. Similarly to what was observed in NeuN-positive cells, a significant rise in the proportion of cells showing this effect was only evident in the HSV-1-EAE group and in the brain, with no apparent changes in spinal cords (Supplementary Fig. 6b). Whether this phenomenon is linked to increased EAE severity or chronic disease course in these animals remains to be evaluated.

**Fig. 7:**
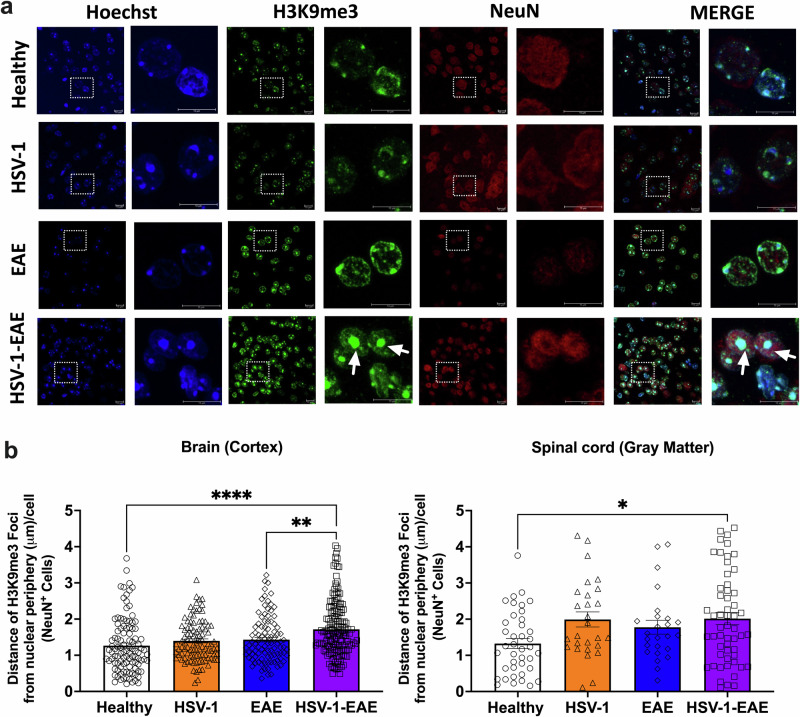
H3K9me3-associated SAHF formation in brain and spinal cord neurons of mice with asymptomatic HSV-1 brain infection and EAE. Brain and spinal cord tissues from four animals per group were harvested 14 days after EAE induction, 45–50 days after HSV-1 infection, or mock-treatment alone for detecting the expression of H3K9me3 foci by immunofluorescence. **a** Representative images showing Hoechst nuclei staining (blue), H3K9me3 staining (green), NeuN marker (red), and image merges. Left: images in each fluorescence channel correspond to 100X magnifications, and right: Images are shown at a 5X optic zoom of the area outlined in squares with white dashed lines. Scale bars = 10 μm. White arrows show senescence-associated heterochromatin foci (SAHF). **b** Quantification of the distance of each H3K9me3 foci from the nuclear periphery in NeuN^+^ cells. Values represent means ± SEM of the measurements carried out in at least ten fields in the brain tissues and three fields in the spinal cord tissues per sample. Data were analyzed using One-way ANOVA followed by Bonferroni’s post-hoc test for analysis from brain tissues and Kruskal–Wallis followed by Dunn’s post-hoc test for analysis from spinal cord tissues; ****p < 0.0001, **p < 0.01, *p < 0.05. White bars with circles represent data from healthy mice, orange bars with triangles represent data from mice with HSV-1 infection, blue bars with diamonds represent data from mice with EAE and purple bars with squares represent data from mice with EAE and HSV-1 infection.

Other consequences of persistent DNA damage, SASP, and senescence growth arrest is chromatin modification and the formation of SAHF^38,39^. SAHF are highly condensed domains of facultative heterochromatin that have been proposed to induce cellular senescence by suppressing the transcription of genes in proliferative cells^40^. However, their expression and relationship with senescence-like phenotypes in post-mitotic neurons remains undefined^23^. Therefore, we investigated their potential involvement in the senescent phenotype observed in neurons during asymptomatic HSV-1 brain infection and/or EAE. At present, there are several molecular markers related to SAHF, such as the histone H2A variant (macroH2A), tri-methylated lysine 9 histone H3 (H3K9me2/3), and heterochromatin protein 1 (HP1), which are characterized by specific physical locations within the nucleus showing chromatin rearrangement at the periphery of this compartment^41^. Here, we evaluated H3K9me3-associated SAHF in neurons (Fig. 7a), and non-neuronal cells (i.e., microglia and OPCs, Supplementary Fig. 7a) in the brain and spinal cord using immunofluorescence and measuring the distance between H3K9me3 foci and the nuclear periphery in NeuN-positive and NeuN-negative cells (Fig. 7b and Supplementary Fig. 7b). Remarkably, analysis performed by an observer blinded to specimen identity showed that SAHF was preferentially formed in neurons of the brain and spinal cord tissues following EAE in mice with previous asymptomatic HSV-1 infection (Fig. 7a). Furthermore, we found a slight, yet significant increase in the mean distance between SAHF foci and the periphery, from 1.3 μm in neurons of healthy mouse brain tissues to 1.7 μm in those from HSV-1-EAE mice. Similarly, in the spinal cord, these measurements were 1.3 and 2.0 μm for healthy and HSV-1-EAE mice, respectively (Fig. 7b). These observations suggest that previous asymptomatic HSV-1 infection in combination with EAE may alter local chromatin interactions leading to changes in physical compaction associated with senescence. However, further studies are needed to determine whether these changes are a contributing factor, or a consequence of exacerbated EAE when having a previous asymptomatic HSV-1 CNS infection.

## Discussion

Although senescence biomarkers in the CNS have been identified in neurodegenerative diseases such as Alzheimer’s disease and Parkinson’s disease^23,42^, the presence of such determinants in these tissues has been poorly explored in the context of viral infections^43^. Similarly, their existence in exacerbated EAE and multiple sclerosis disease has been reported somewhat limitedly^12,33,44^. While a seminal study reported lipofuscin accumulation, a marker of cellular senescence, in brain lesions of multiple sclerosis patients, it is worth noting that all the tissues analyzed in the corresponding study were obtained from individuals aged 70 years or older. Consequently, the study could not differentiate whether the presence of lipofuscin was primarily associated with the aging process or specifically attributed to multiple sclerosis^12^. Hence, there is an urgent need for research aimed at providing additional evidence linking cellular senescence to the pathogenesis of multiple sclerosis in such a way as to identify novel treatment options.

Here, we found that asymptomatic HSV-1 brain infection elicits a significant expression of numerous senescence biomarkers in the CNS, namely increased p21- and p53-related mRNA levels and elevated SASP-associated mRNA levels related to cytokines, such as IL-6 and MMP-12. Importantly, we also found that after latent infection of the brain with HSV-1 and EAE, there was an elevated expression of specific senescence biomarkers in the CNS. Taken together, these data suggest that a previous asymptomatic HSV-1 brain infection is associated with a significant increase in the expression of senescence biomarkers in the CNS, particularly associated with DNA damage in neurons, which could perpetuate a proinflammatory environment permissive for the initiation, or worsening of autoimmunity as proposed for other diseases, such as Parkinson’s and systemic lupus erythematosus^42,45^.

The results described herein complement previous findings where the accumulation of DNA damage was reported in cortical neurons in vitro after productive infection with HSV-1. These results suggest a role for this virus in the development of neurodegenerative disorders through the accumulation of CNS injuries due to deficient host DNA repair^46^. Importantly, we found that DNA damage was observable in neurons during EAE, with or without previous asymptomatic HSV-1 brain infection, as detected by increased γHA2X foci in these cells. Interestingly, a recent study reported that p53 and H2AX proteins bind to the promoters of senescence-related genes and regulate their expression, further relating DNA damage to cellular aging^42^. Moreover, another study showed that DNA damage in oligodendrocytes results in the induction of a senescent-like phenotype in these cells, leading to progressive CNS demyelination, reminiscent of progressive multiple sclerosis in a mouse model, and the inhibition of the p53 pathway via p21 decreased disease severity^47^. In line with this observation, another study reported γHA2X staining in the trigeminal ganglia during acute HSV-1 infection in mice, reinforcing the notion that this virus elicits DNA damage in neurons, or at least elicits these molecular markers in these cells^48^. Noteworthy, to our knowledge, the presence of DNA damage-related markers in neurons during viral latency or in the CNS of mice infected with HSV-1 has not been reported before, which deserves further studies given that impaired DNA damage responses (DDRs) have been widely documented in several neurodegenerative disorders^42,49^. Indeed, a study proposed that the expression of γH2AX in peripheral blood mononuclear cells (PBMCs) could be a potential diagnostic tool and a marker for disease activity in relapsing-remitting multiple sclerosis (RRMS)^50^. This conclusion was drawn from the observation that individuals with RRMS display elevated levels of γH2AX-positive cells compared to healthy controls. Additionally, the levels of γH2AX-positive cells correlate with magnetic resonance imaging (MRI) indicators of disease activity in RRMS patients^50^. However, a subsequent study showed conflicting results, as no significant differences were observed regarding the levels of γH2AX and 53BP1 foci between multiple sclerosis patients and healthy individuals. Furthermore, there was no discernible relationship between the levels of γH2AX and 53BP1 and the MRI indicators^51^.

Importantly, our findings support a previous study performed in a mouse model of HSV-1 eye infection without scarification that reported increased mRNA levels of senescence-associated genes encoding p16, p53, NLR family pyrin domain containing 3 (NLRP3), IL-1α, and IL-1β proteins in the brainstem of HSV-1-latently infected female mice, as opposed to male mice, hence our preference for using female mice in this study, together with the fact that male mice undergo less EAE^52,53^. However, a detailed characterization of senescence-associated markers in specific cell types was not carried out in that study, and more in-depth analyses at the protein level were missing^52^. Here, we identified a distinctive senescent phenotype, particularly in neurons after intranasal HSV-1-inoculation and later EAE in female mice, characterized by SAHF in the brain and the spinal cord. Remarkably, we also detected several senescence-related markers in the spinal cord of animals asymptomatically infected with HSV-1 alone. These findings suggest that viral access to the spinal cord tissue early after intranasal infection may be sufficient to trigger neuroinflammation and DNA damage in neurons at this site, which is supported by our qPCR analyses. In this regard, a previous study reported that footpad inoculation of another herpesvirus, namely pseudorabies virus was sufficient to activate dorsal root ganglia (DRG) neurons of the spinal cord to produce cytokines^54^. Importantly, previous studies have also shown that viral infections can increase the susceptibility to autoimmune diseases by eliciting bystander inflammation, which lowers the threshold for disease onset^55,56^. This notion is also consistent with our previous observation that a mutant HSV-1 virus that is attenuated in establishing latency and reactivating from neurons also elicits enhanced EAE after inoculation, suggesting that more severe forms of EAE could be mediated by inflammatory signatures imprinted to the CNS early after infection and do not necessarily depend on viral reactivation or persistence^11^. It will be interesting to determine which of the markers associated with senescence identified in our study may play a prominent role in EAE exacerbation observed after previous asymptomatic HSV-1 brain infection and potential approaches that may be used to modulate EAE outcome favorably.

An important finding was that HMGB-1 emerged as an alarmin in brain tissues, specifically in mice with HSV-1 infection and EAE. Notably, a proteomic analysis of the secretome of neural progenitor cells derived from induced pluripotent stem cell lines obtained from patients with primary progressive multiple sclerosis found elevated levels of HMGB-1 associated with reduced oligodendrocyte maturation^57^. A subsequent study by the same group reported that HMGB-1 directly inhibits the differentiation of oligodendrocytes progenitor cells and impairs CNS remyelination^34^. Hence, it is possible to consider that impaired remyelination processes could be induced in mice with asymptomatic HSV-1 brain infection and EAE due to the chronic release of HMGB-1 in the CNS. Our findings also align with previous studies reporting increased HMGB-1 expression during EAE progression^58^. Interestingly, one study reported that an anti-HMGB-1 monoclonal antibody attenuates the progression of EAE^59^. In addition, a recent study showed higher plasma levels of HMGB-1 in multiple sclerosis patients compared to healthy individuals, which highly correlated with the number of affected zones in the brain^33^.

Additionally, recent studies support the notion that remyelination is regulated by age-dependent epigenetic control of gene expression, which in turn is associated with the recruitment of histone deacetylases (HDACs)^60,61^. Indeed, in demyelinated aged brains, the recruitment of HDACs is inefficient, possibly contributing to impaired remyelination. Moreover, HDAC inhibitors in mice have been shown to recapitulate defective remyelination, offering a compelling link between epigenetic regulation and myelin repair^62^. Notably, a recent study showed that both in vitro and in vivo recurrent HSV-1 infection increase H4K16ac, Sin3, and HDAC1 levels, which have been related to the aging process in several organisms^63^. Regarding the EAE model, promising results were observed upon using the HDAC inhibitor belinostat, which elicited a reduction in the severity of EAE in mice, primarily attributed to its capacity to inhibit Toll-like receptor-2/Myeloid differentiation primary response 88 (TLR2/MyD88)- and HDAC3/NF-κB-p65-mediated neuroinflammation^64^. Another therapeutic candidate explored in the EAE model is a novel HDAC6-selective inhibitor that displays both prophylactic and therapeutic effects by modulating peripheral immune responses and preserving the integrity of the blood-brain barrier (BBB)^65^. Importantly, it has been described that unbalanced epigenetic dynamics could lead to cancer, neurodegeneration, and premature aging^66^. Our findings support the notion that a previous HSV-1 infection may trigger a specific neuronal senescence-like phenotype in individuals undergoing an autoimmune condition in the CNS. Indeed, we observed in the mice an increase in H3K9me3 SAHF formation in neurons. Notably, disease outcome could be influenced by changes in the chromatin landscape given by the interplay between environmental and host factors.

On the other hand, DNA methylation has long been suspected to play a significant role in immune processes that trigger and enhance multiple sclerosis pathogenesis^17,66–68^. Consistent with this notion, reduced inflammation and disease severity were elicited by others by inhibiting all DNA methylation reactions with methylthioadenosine (MTA) in a rat model of EAE^69^. Based on these and other related findings, there is growing interest in the potential therapeutic exploitation of chromatin modulation to moderate neurodegenerative diseases^60,65,70^. In this sense, therapies targeting heterochromatin alterations, such as those possibly evidenced by H3K9me3 foci suggesting HSV-1-induced premature senescence-like state in neurons could hold promise in treating chronic brain damage induced by latent viral CNS infection. However, further studies are needed to identify if chromatin alterations such as H3K9me3 play a relevant role in multiple sclerosis exacerbation.

Overall, the findings reported herein warrant additional studies on CNS senescence induced by asymptomatic HSV-1 brain infection and EAE to better define possible relationships between CNS infection by this persistent virus and neurodegenerative diseases such as multiple sclerosis. However, to better understand multiple sclerosis pathology, a combination of experimental models, including other EAE models alongside clinical studies, will be necessary for capturing the overall complexity of the disease and envisaging new effective therapeutic strategies. While the EAE model used herein provides valuable insights into certain aspects of multiple sclerosis, it nevertheless has some limitations in fully recapitulating all aspects of multiple sclerosis, such as the lack of the chronic and progressive nature of multiple sclerosis, and it focuses on an immune response based on CD4^+^ T cells that are mainly directed to the spinal cord, while multiple sclerosis is primarily driven by CD8^+^ T cells^71^. Nevertheless, our study sheds light on senescence processes occurring in the brain and spinal cord upon EAE, which is deemed to play significant roles in multiple sclerosis pathology in humans. Moreover, while HSV-1 infection has been extensively studied for its effects on brain alterations through direct and indirect mechanisms, its effects on the spinal cord has received comparatively lesser attention. Given our findings, not only the presence of HSV-1 material in the spinal cord is likely to have direct effects on this tissue, but it is also plausible that upon asymptomatic brain infection with HSV-1 inflammation elicited by the infection at this site extends on to distant tissues, including the spinal cord, potentially contributing to alterations at this site that may contribute to demyelinating pathologies such as multiple sclerosis. Hence, future studies in this line should ideally also include additional EAE animal models, such as the cuprizone mouse model and the lysolecithin-induced demyelination model to facilitate a deeper understanding of the influence of HSV-1 infection, as well as senescence on demyelination and remyelination processes.

Finally, it will be of great importance to assess the effects of drugs targeting senescence, such as senotherapeutics over HSV-1-induced senescence in the CNS with or without EAE for elucidating potential new therapeutic interventions that could mitigate the impact of senescence elicited by viral infection over CNS function and its contribution to neurodegenerative diseases like multiple sclerosis^72^.

## Materials and methods

### Mice

Female C57BL/6 mice aged five weeks were sourced from The Jackson Laboratory (Bar Harbor) and were housed at the central animal facility of Pontificia Universidad Católica de Chile. They were provided environmental enrichment, sterile food, and water ad libitum. The protocols employed in this study were approved by the Scientific Ethical Committee for Animal and Environmental Care of Pontificia Universidad Católica de Chile and the institutional Biosafety Committee (Protocols #170705018 and #210425003). All procedures were conducted following the National Institutes of Health Guide for the Care and Use of Animals^73^.

### Virus propagation

Vero cells (ATCC CCL-81) were used to propagate and titer the HSV-1 strain 17syn^+^. Briefly, T175 flasks with Vero cells monolayers were inoculated with HSV-1 at a multiplicity of infection (MOI) 0.01 in 20 mL of Opti-MEM (Gibco, Life Technologies) and incubated at 37 °C for 48 h until visible cytopathic effect. Then, the content of the flasks was pooled, and cell debris was removed twice by centrifugation at 15,000×*g* for 10 min. The pellet was resuspended with 1 mL of Opti-MEM and then sonicated in an ultrasonic bath for 10 min with pulses of 10 s. These stocks were then stored at −80 °C until required. The WT HSV-1 (17syn^+^ strain) was kindly provided by Dr. Carola Otth from Universidad Austral de Chile, Chile.

### Infections and EAE induction

Female C57BL/6 mice were intranasally infected with a sub-lethal dose of 10^6^ plaque-forming units (PFU) of WT HSV-1 strain 17syn^+^, as described in previous studies^11,74^. Mock-treated mice were utilized as control subjects and received an inoculation of supernatant derived from Vero cell cultures. Over the initial two weeks following infection, the mice were evaluated daily for neurological symptoms using the following scoring system: Normal (0), ataxia (1), hunched posture (1), forelimbs paralyzed yet mobile-capable (1), forelimbs paralyzed and immobile (2), seizures or circling (1). The scores for each symptom were aggregated to generate the final cumulative clinical score. This experimental approach induced asymptomatic HSV-1 infection without manifesting clinical signs of encephalitis, in line with previous reports^11^. A mild form of EAE was induced in the mice 30–35 days post-asymptomatic HSV-1 infection to evidence any potential increase in the severity of EAE caused by prior asymptomatic HSV-1 infection. This model and related data were previously reported in Duarte et al., 2021 and the main findings related to this article are summarized in Supplementary Fig. 1 included herein under permission granted by CC-BY Creative Commons attribution license used by the publisher of the indicated article^11^. Mice were anesthetized using a combination of ketamine (80 mg/kg) and xylazine (4 mg/kg), injected subcutaneously with 50 µg of myelin oligodendrocyte glycoprotein (MOG)-derived peptide (MOG_35 − 55_, sequence MEVGWYRSPFSRVVHLYRNGK; Pan Web, Stanford University). The peptide was emulsified in complete Freund’s adjuvant (Thermo Fisher Scientific) supplemented with heat-inactivated Mycobacterium tuberculosis H37 RA (DIFCO). Additionally, mice received two intraperitoneal injections of 350 ng of pertussis toxin (List Biological Laboratories, Inc.) at the time of induction and 48 h later. Daily assessments of motor function were conducted using the EAE scale: 0, no changes; 0.5, limp tail tip; 1, limp tail; 2, limp tail and hind leg weakness; 2.5, limp tail and paralysis of one hind limb; 3, limp tail and complete hind limb paralysis; 3.5, paralysis of hind limbs and one forelimb; 4, complete paralysis of hind limbs and forelimbs; 5, moribund.

### Quantitative PCR (qPCR)

Total DNA was isolated from brains, spinal cords, and trigeminal ganglia tissues at days 4 and 30 after HSV-1 infection using TRIzol reagent (Thermo Fisher Scientific) according to the manufacturer’s instructions. Two hundred nanograms (200 ng) of DNA were used for qPCR analyses with the following primers and probe for the *UL30* gene (HSV-1 polymerase): Fwd-GGCCAGGCGCTTGTTGGTGTA, Rev-ATCACCGACCCGGAGAGGGA and Probe-CCGCCGAACTGAGCAGACACCCGC (Integrated DNA Technologies), as previously described in ref. ^75^. Tenfold dilutions of purified HSV-1 DNA contained within a bacterial artificial chromosome (BAC)^76^ were used to generate a standard curve of known numbers of viral genome copies with the corresponding CT values. Then, CT readouts obtained for each tissue sample (brain, spinal cord, and trigeminal ganglia) were interpolated into this curve to determine absolute viral genome copy numbers. The limit of detection of the method was >26 viral genome copies per 200 ng of extracted DNA, based on samples obtained from each type of tissue of the mock-treated group.

### Reverse transcription-quantitative PCR (RT-qPCR)

Total RNA was isolated from brain and spinal cord tissues for gene expression analyses at days 14 and 21 post-EAE induction or days 45 and 52 post-HSV-1 infection using TRIzol reagent (Thermo Fisher Scientific) according to the manufacturer’s instructions. RT-qPCR reactions were carried out using TaqMan® RNA-to-Ct^TM^ 1-Step Kit (Thermo Fisher Scientific) and TaqMan® probes for the detection of *Hmgb1* (Ref: Mm00849805_gH), *Cdkn1a* (Ref: Mm04205640_g1), *Cdkn2a* (Ref: Mm00494449_m1), *Pdrg1* (Ref: Mm00724869_m1), *Lmnb1* (Ref: Mm00801853_m1), Timp1 (Ref: Mm01341361_m1), *Mmp12* (Ref: Mm00500554_m1), *Cxcl2* (Ref: Mm00441242_m1), *Ccl2* (Ref: Mm00441242_m1), *Il-6* (Ref: Mm00446190_m1), and *β-actin* (Ref: Mm02619580_g1) on the StepOnePlusTM Real-Time PCR System (Applied Biosystems R) with the following cycling conditions: one cycle at 50 °C for 15 min and 95 °C for 10 min, followed by 40 cycles at 95 °C for 15 s, and 60 °C for 1 min. The abundance of each target mRNA was determined by relative expression to the *β-actin* housekeeping gene using the 2^−ΔΔCT^ method^77^.

### Detection of senescence-associated beta-galactosidase (SA-β-Gal)

Brain and spinal cord tissues were embedded in a tissue-freezing medium (OCT, Sakura). Frozen tissue sections (5 µm) were fixed with 2% w/v paraformaldehyde and 0.2% v/v glutaraldehyde for 5 min at room temperature. After three 5-min washes with PBS, sections were immersed in a staining solution consisting of 40 mM citric acid/Na phosphate buffer at pH 6.0, 5 mM K_4_[Fe(CN)_6_] × 3H_2_O, 5 mM K_3_[Fe(CN)_6_], 150 mM NaCl, 2 mM MgCl_2_, and 1 mg/mL X-gal (Bioline, Taunton, MA, USA) for 24 h at 37 °C.

### Immunohistochemistry

Mice subjected to HSV-1 infection and EAE induction were subjected to transcardial perfusion with 50 mL of PBS to eliminate intravascular leukocytes. At days 14 and 21 post-EAE induction or days 45 and 52 post-HSV-1 infection, spinal cords and brains were meticulously dissected and processed for histological assessments. The tissues were initially fixed in 4% PFA for 24 h, followed by ethanol dehydration and paraffin embedding. Sections were then deparaffinized using xylene and gradually rehydrated using alcohol with decreasing concentrations. Antigen retrieval was performed as reported previously in ref. ^78^. Briefly, tissue samples were incubated in a buffer containing Tris-EDTA (pH=9) at 80 °C for 30 min. Subsequently, the slides underwent treatment with a 3% v/v hydrogen peroxide solution for 20 min, followed by blocking with 10% v/v calf serum and 0.3% v/v Triton X-100 in PBS for 2 h. The samples were then exposed to overnight incubation with anti-γH2AX antibody (Millipore #05-636) at 4 °C. Negative control sections were treated with non-immune calf serum instead of the primary antibody. After three 10-min washes, the primary antibody was detected through incubation with anti-mouse/rabbit IgG RTU biotinylated secondary antibody (Vector Laboratories, Burlingame, CA, USA, catalog #BP-1400) for 30 min at room temperature. Amplification of secondary antibody labeling was achieved using the ABC Peroxidase Standard Staining Kit (Thermo Scientific catalog #32020) for 30 min at room temperature. Following three PBS washes, the immunolabeling signal was visualized using DAB Peroxidase substrate (Vector Laboratories catalog #SK-4100). Luxol Fast Blue (0.1%, 2 h at 56 °C) was employed for counterstaining. Images were captured using a Nanozoomer-XR digital slide scanner C12000 (Hamamatsu Photonics KK, Hamamatsu, Japan) after completing the immunohistochemical procedures.

### Immunofluorescence

Antigen retrieval was performed by incubating the tissue samples with a buffer containing Tris-EDTA pH 9 for 30 min at 80 °C. The slides were then blocked with 10% v/v fetal bovine serum and 0.3% v/v Triton X-100 diluted in PBS pH 7.4 for 2 h. The samples were then incubated with either anti-NeuN (Abcam, ab177487, 1:200 dilution), anti-IBA-1 (Cell Signaling #17198, 1:200 dilution), anti-A2B5 (Sigma, MAB312, 1:200 dilution), anti-HMGB1 (R&D system, MAB1690, 1:100 dilution), anti-H3K9me3 (Novusbio # 6F12-H4, 1:200 dilution), anti-phospho histone H2AX (Ser139) (Millipore #05-636, 1:300 dilution), or anti-53BP1 (Novusbio # 100-304, 1:200 dilution) overnight at 4 °C. For negative control sections, the primary antibody was replaced by the blocking solution. After being washed three times for 10 min each, the primary antibodies were detected by incubation in an anti-secondary isotype-specific conjugate with Alexa Fluor at room temperature. Then, sections were washed three times with PBS Tween 20 0.05% v/v, pH 7.4, and autofluorescence was quenched using a Quenching kit (Vector laboratories #SP-8400). Finally, sections were stained with Hoechst for 10 min and mounted with anti-fade mounting media (Vector #30304) according to the manufacturer’s recommendations. Images were acquired using a Leica SP8 confocal microscopy at 100x magnification. The images were processed using Leica Application Suite X (LAS X) software and analyzed using FIJI-ImageJ software (US National Institutes of Health)^79,80^.

### Statistics and reproducibility

Means and standard error of the mean (SEM) were reported as summary statistics. Statistical analyses between experimental groups were assessed using either unpaired Student’s *t*-test (for two groups) and one-way analysis of variance (ANOVA) (for three groups) followed by Dunnett’s post-hoc test or Bonferroni´s correction for multiple comparisons when mean values could be assumed normal, or nonparametric Kruskal–Wallis test followed by Dunn’s post-hoc test when normality could not be assumed. Two-way ANOVA with Tukey’s multiple comparison post-hoc test was used for analyzing two independent variables. The parameters evaluated among groups and combined illnesses with infection conditions (healthy, EAE, HSV-1, or HSV-1-EAE) are indicated in the figure legends. Clinical EAE scores from day 0 to 25 comparing the EAE vs HSV-1-EAE groups was done using the Multiple Mann–Whitney test, with the area under the curve (AUC) used to quantify the overall effect. The significance level was set at 0.05. Statistical analyses were conducted using GraphPad Prism Version 9.4.1. (GraphPad Software).

### Reporting summary

Further information on research design is available in the Nature Portfolio Reporting Summary linked to this article.

### Supplementary information

Supplementary Information

Description of Additional Supplementary Files

Supplementary Data 1

Reporting Summary

## Data Availability

All data generated during this study are included in the published article and supplementary files. The source data behind the graphs in the paper can be found in Supplementary Data 1. Additional inquiries can be directed to the corresponding authors.
