# Supplementary material for: Asymptomatic herpes simplex virus brain infection elicits cellular senescence phenotypes in the central nervous system of mice suffering multiple sclerosis-like disease
Source: Commun Biol. 2024 Jul 4;7:811. doi: 10.1038/s42003-024-06486-x (PMC11224417; doi:10.1038/s42003-024-06486-x)
Supplement: Supplementary file 1 — Supplementary Information [file 42003_2024_6486_MOESM1_ESM.pdf]

# ASYMPTOMATIC HERPES SIMPLEX VIRUS BRAIN INFECTION ELICITS CELLULAR SENESCENCE PHENOTYPES IN THE CENTRAL NERVOUS SYSTEM OF MICE SUFFERING MULTIPLE SCLEROSIS-LIKE DISEASE

**Luisa F. Duarte[1,2], Verónica Villalobos[1,3], Mónica A. Farías[1,4], Ma. Andreina Rangel-Ramírez[1,2], Enrique González-Madrid[1,2], Areli J. Navarro[1,4], Javier Carbone-Schellman[1,4], Angélica Domínguez[5], Alejandra Alvarez[1,4], Claudia A. Riedel[1,2], Susan M. Bueno[1,4], Alexis M. Kalergis[1,4,6], Mónica Cáceres[1,3,*] and Pablo A. González[1,4,*]**

[1]Millennium Institute on Immunology and Immunotherapy, Santiago, Chile.

[2]Departamento de Ciencias Biológicas, Facultad de Ciencias de La Vida, Universidad Andrés Bello, Santiago, Chile.

[3]Program of Cellular and Molecular Biology, Institute of Biomedical Sciences, Faculty of Medicine, Universidad de Chile, Millennium Nucleus of Ion Channel-Associated Diseases (MiNICAD), Santiago, Chile.

[4]Facultad de Ciencias Biológicas, Pontificia Universidad Católica de Chile, Santiago, Chile.

[5]Departamento de Salud Pública, Facultad de Medicina, Pontificia Universidad Católica de Chile, Santiago, Chile.

[6]Departamento de Endocrinología, Facultad de Medicina, Pontificia Universidad Católica de Chile, Santiago, Chile.

*Correspondence: Dr. Pablo González, Millennium Institute on Immunology and Immunotherapy, Facultad de Ciencias Biológicas, Pontificia Universidad Católica de Chile, Av. Portugal 49, Santiago E-8330025, Chile. E-mail: pagonzam@uc.cl and Dr. Mónica Cáceres, Millennium Institute on Immunology and Immunotherapy, Instituto de Ciencias Biomédicas, Facultad de Medicina, Universidad de Chile, Independencia 1027 Santiago,Chile. E-mail: monicacaceres@med.uchile.cl

## SUPPLEMENTARY FIGURES

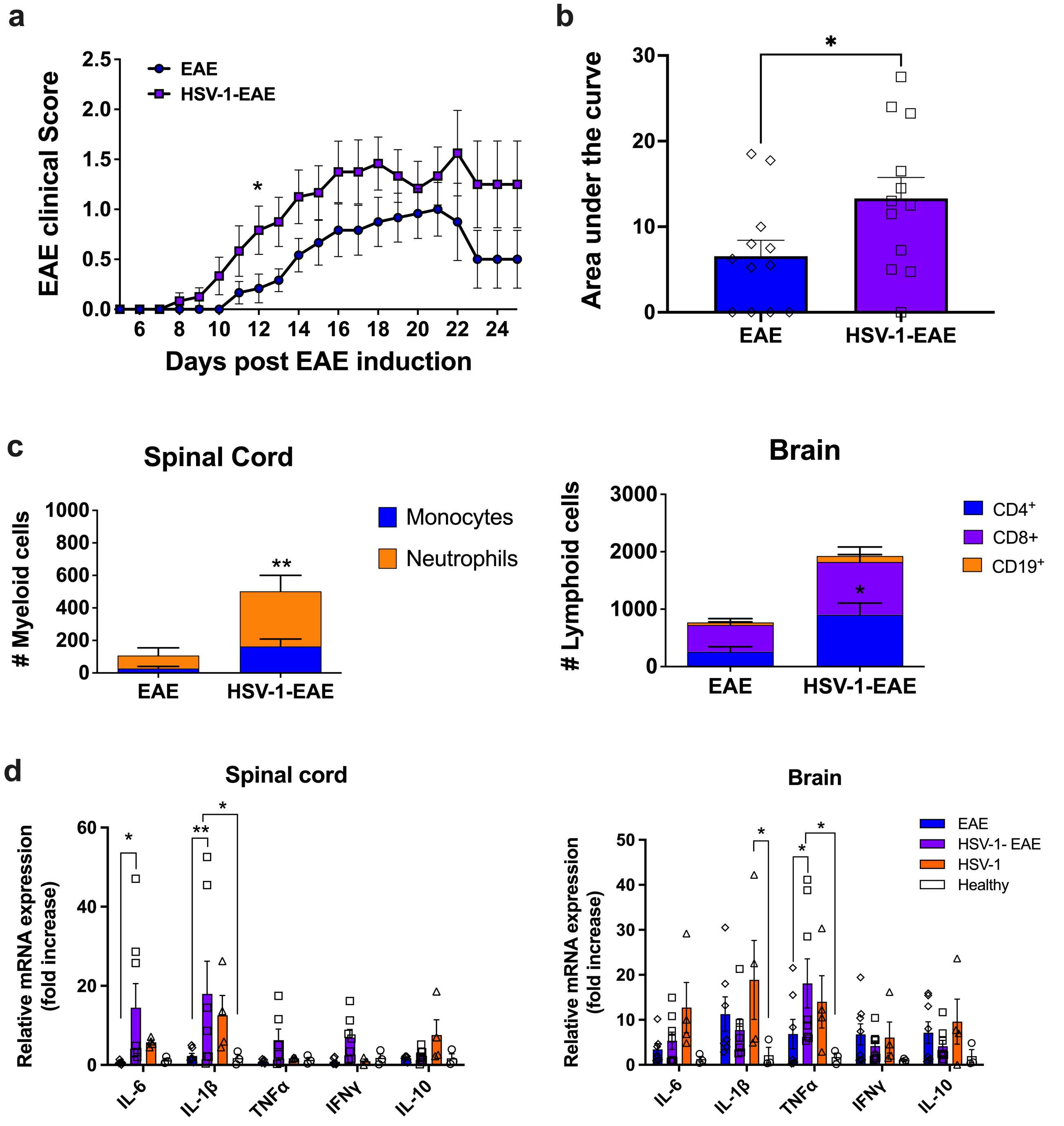


**Supplementary figure 1: Asymptomatic HSV-1 Infection causes an earlier onset and more severe EAE. a** EAE clinical score was plotted for 12 mice after EAE induction. EAE was induced 30–35 days post-HSV-1 asymptomatic brain infection. The graph shows the means of disease scores ±

SEM for mice mock-treated (blue circles) or infected with WT HSV-1 ($17syn^{+}$ strain, purple squares). The data were analyzed using Multiple Mann-Whitney test, *$p<0.05$. **b** The area under the curve (AUC) integrates the clinical EAE scores and their duration between days 0-25. Data shown are means ± SEM of three independent experiments. Blue bars with diamonds represent data from mice with EAE and purple bars with squares represent data from mice with EAE and HSV-1 infection. The data were analyzed using an unpaired two-tailed Student's t-test, *$p<0.05$. **c** Graphs show the whole number of infiltrating myeloid cells in the spinal cord (left panel) and infiltrating lymphoid cells in the brain (right panel) of mice induced to develop EAE with or without previous HSV-1 infection. Data are means ± SEM of two independent experiments n=8 animals/group. The data were analyzed using Two-way ANOVA followed by Bonferroni's post-test, **$p<0.01$ *$p<0.05$. **d** Relative levels of mRNA of genes encoding pro-inflammatory cytokines IL-6, IL1-β, TNF-α, and IFN-γ, and the anti-inflammatory cytokine IL-10 in the spinal cord (left panel) and the brain (right panel) of HSV-1-infected mice with or without EAE, compared to the mock-EAE group. Blue bars with diamonds represent data from mice with EAE, purple bars with squares represent data from mice with EAE and HSV-1 infection, orange bars with triangles represent data from mice with HSV-1 infection without EAE, and white bars with circles represent data from healthy mice. Values represent means ± SEM of two independent experiments (n=8 animals/group). Data were analyzed using two-way ANOVA followed by Tukey's post-test; **$p<0.01$, and *$p<0.05$. The data presented in this figure was previously published in Duarte *et al.,* 2021 available at doi:10.3389/fimmu.2021.635257. Data from **a-d** were modified from Duarte *et al.,* 2021 to provide a summary and more focused information for this study. Permission to use these figures is granted by CC-BY Creative Commons attribution license used by the publisher.

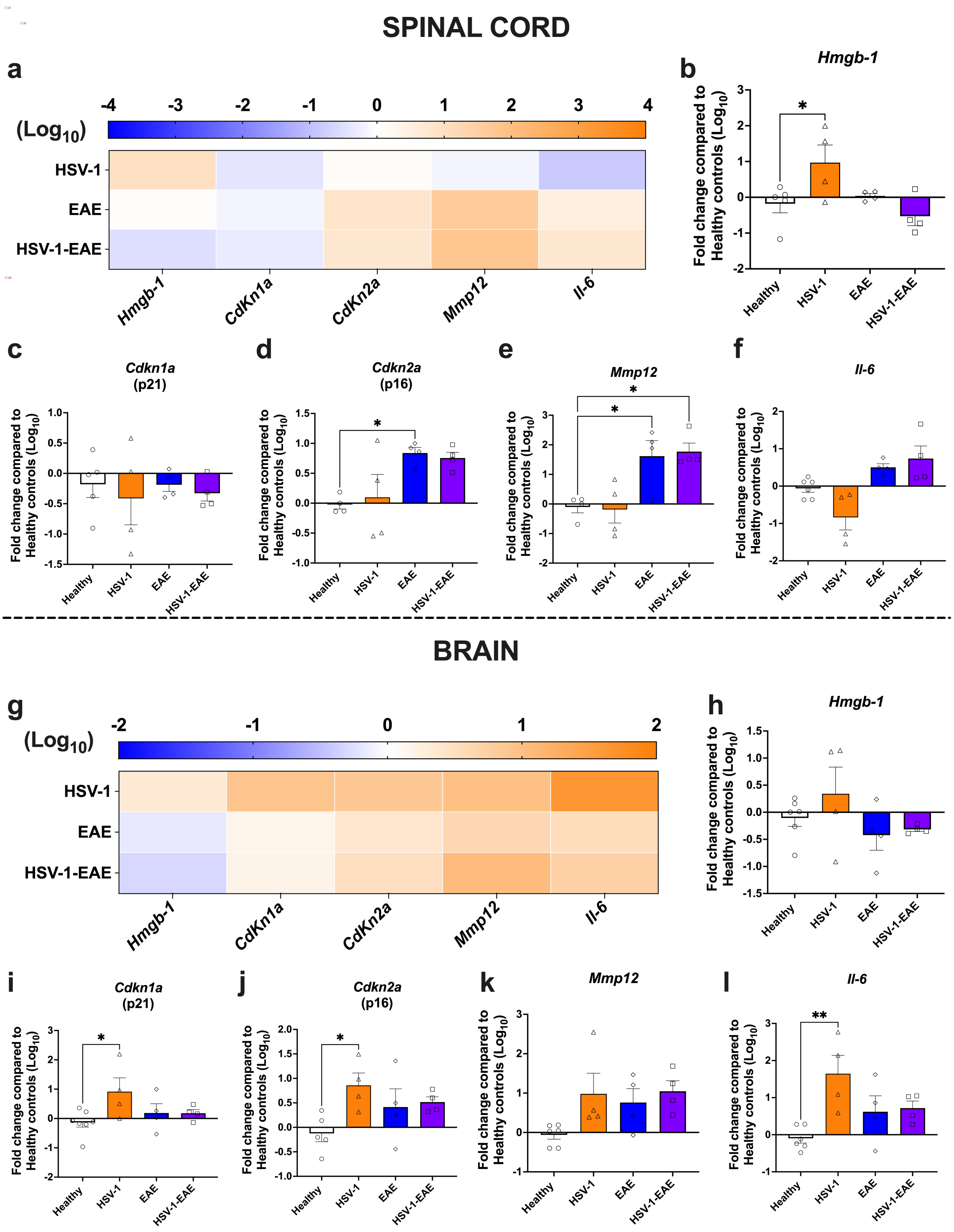
SPINAL CORD
a
(Log10)
-4
-3
-2
-1
0
1
2
3
4
HSV-1
EAE
HSV-1-EAE
Hmgb-1
CdKn1a
CdKn2a
Mmp12
Il-6
b
Hmgb-1
Fold change compared to Healthy controls (Log10)
Healthy
HSV-1
EAE
HSV-1-EAE
c
Cdkn1a (p21)
d
Cdkn2a (p16)
e
Mmp12
f
Il-6
BRAIN
g
(Log10)
h
Hmgb-1
i
Cdkn1a (p21)
j
Cdkn2a (p16)
k
Mmp12
l
Il-6

**Supplementary figure 2: mRNA levels of senescence-associated genes in the CNS increase 21 days after EAE induction regardless of prior HSV-1 infection.** Mice were mock-treated (healthy group, white bars with circles) or asymptomatically infected with HSV-1 strain 17syn$^{+}$ in the brain (HSV-1 group, orange bars with triangles). EAE was induced four weeks after mock treatment (EAE group, blue bars with diamonds), or HSV-1 infection (HSV-1-EAE group, purple bars with squares). Spinal cord and brain homogenates were recovered 21 days after EAE induction (EAE and HSV-1-EAE groups) or 52 days after HSV-infection (HSV-1 group). The expression of senescence-associated genes was evaluated at the mRNA level by RT-qPCR by using the $2^{-\Delta\Delta CT}$method with *Actb* as a reference gene (β-actin protein). **a** Heatmap comparing mRNA levels of senescence-associated genes in the spinal cord. Orange color indicates upregulation, while blue color indicates downregulation. Stronger colors indicate stronger effects. Relative mRNA expression of the gene products **b** *Hmgb-1*, **c** *Cdkn1a*, **d** *Cdkn2a,* **e** *Mmp12, and* **f** *Il6* in the spinal cord homogenates of the different mouse groups compared to healthy controls. **g** Heatmap comparing mRNA levels of senescence-associated genes in the brain. Orange color indicates upregulation, while blue color indicates downregulation. Stronger colors indicate stronger effects. Relative mRNA expression of the gene products **h** *Hmgb-1*, **i** *Cdkn1a*, **j** *Cdkn2a,* **k** *Mmp12,* and **l** *Il6* in the brain homogenates of the different mouse groups compared to healthy controls. Values represent means ± SEM of 4 animals/group. Log-transformed data were analyzed using One-way ANOVA followed by Dunnett's post-test; **p<0.01, and *p<0.05.

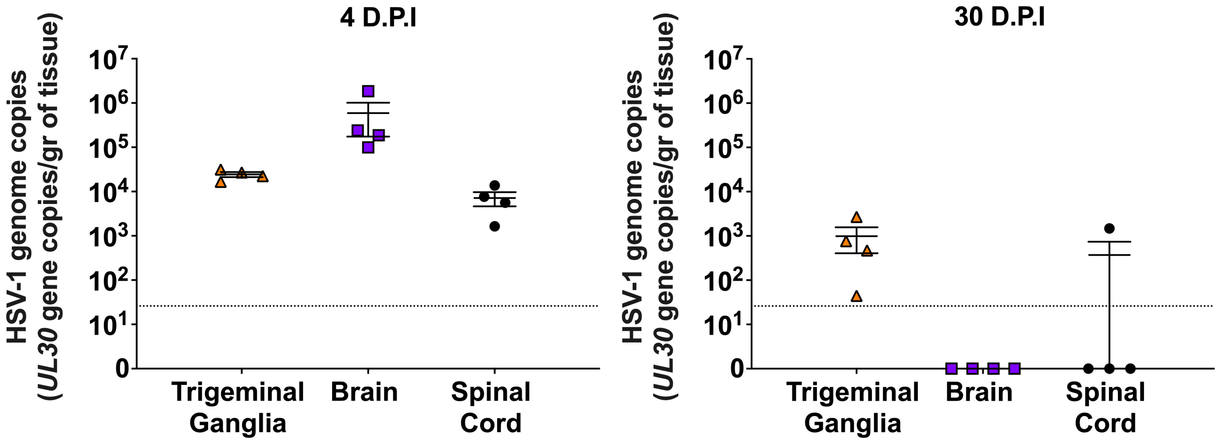


**Supplementary figure 3: Viral loads in the nervous system after sublethal asymptomatic infection with HSV-1 by intranasal route.** C57BL/6 mice were intranasally mock-treated or infected with HSV-1 (17syn+ strain) and followed for 30 days**.** HSV-1 *UL30* gene copies per gram of trigeminal ganglia (orange triangles), brain (purple squares) and spinal cord (black circles) was determined at 4- and 30-days post-infection by qPCR (values normalized to uninfected mice, n=4 animals/group). Data were analyzed using One-way ANOVA followed by Dunnett's post-test. There were no statistically significant differences between groups. Dashed lines indicate the limit of detection of viral genome copies (>26 viral genome copies per 200 ng of extracted DNA) based on samples obtained from each type of tissue from the mock-treated group.

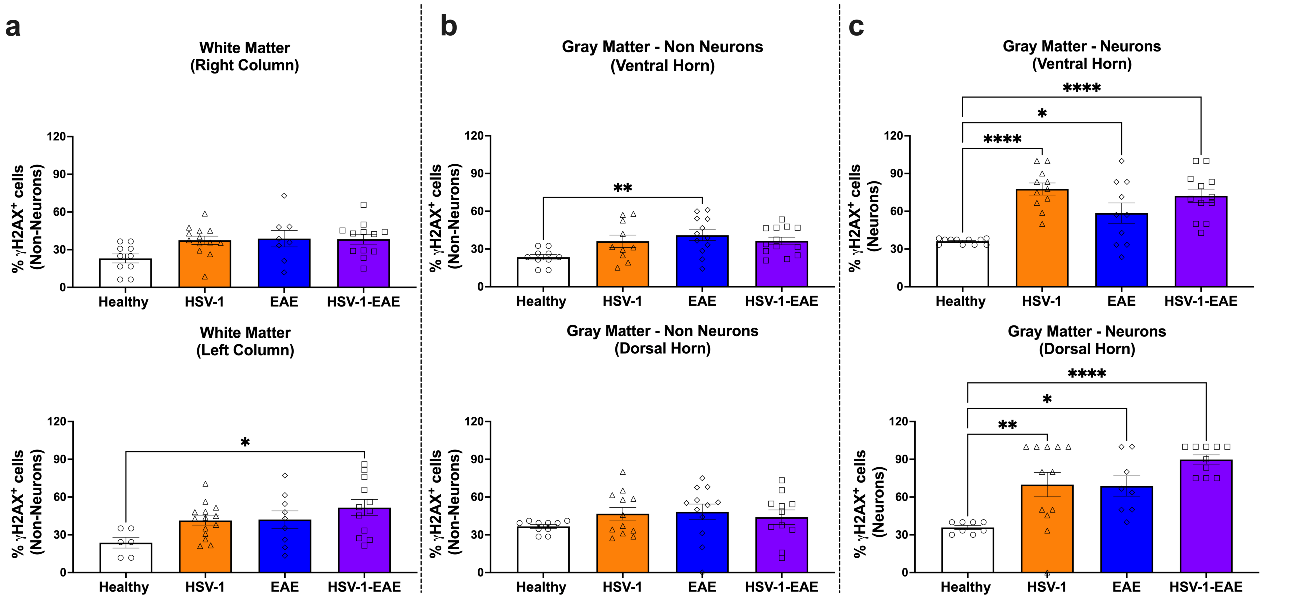


**Supplementary figure 4: DNA damage-related foci are observed in neurons and non-neurons cells 21 days after EAE induction in the spinal cord tissues.** Spinal cord tissue was harvested 21 days after EAE induction (EAE and HSV-1-EAE groups) and 52 days after HSV-1 infection (HSV-1 group) for detecting the phosphorylation of histone H2AX (γH2AX) by immunohistochemistry. **a** Quantification of γH2AX foci in the nucleus of non-neuron cells in the white matter (analysis separated in right and left columns, upper and lower panels, respectively). **b** Quantification of γH2AX foci in the nucleus of non-neuron cells in the gray matter (analysis separated in ventral and dorsal horns, upper and lower panels, respectively). **c** Quantification of γH2AX foci in the nucleus of neuron cells in the gray matter (analysis separated in ventral and dorsal horns upper and lower panels, respectively). Values represent means ± SEM of the percentage of γH2AX-positive cells of four mice per group. Data were analyzed using One-way ANOVA followed by Bonferroni's post-test; ****p<0.001, **p<0.01, *p<0.05. White bars with circles represent data from healthy mice, orange bars with triangles represent data from mice with HSV-1 infection, blue bars with diamonds represent data from mice with EAE and purple bars with squares represent data from mice with EAE and HSV-1 infection.

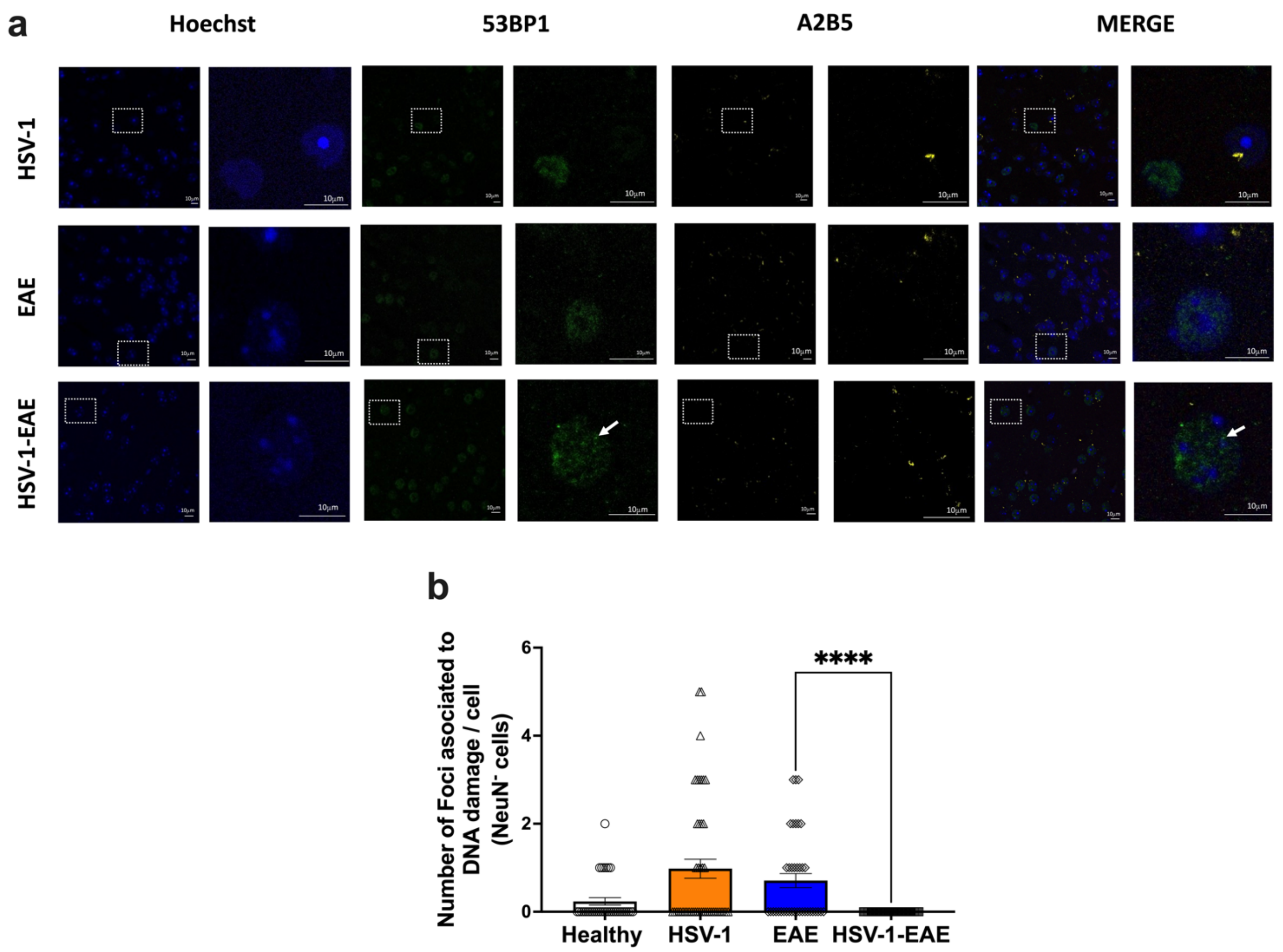


**Supplementary figure 5: DNA damage-related foci are rare or absent in non-neuronal cells within the brain of mice with asymptomatic HSV-1 brain infection and/or EAE.** Brain tissues were harvested 14 days after EAE induction or 45 days after asymptomatic HSV-1 brain infection, or mock treatment alone for detecting the 53BP1 protein which is used as a DNA damage marker by immunofluorescence. **a** Representative images showing Hoechst nuclei staining (blue color), 53BP1 straining (green color), A2B5 staining (yellow color, oligodendrocyte progenitor marker), and image merges. Left: images for each fluorescence channel correspond to 100X magnifications and right: images are shown at a 5X optic zoom of the area outlined in squares with white dashed lines. Scale bars = 10 μm. White arrows show accumulation of 53BP1 into DNA damage foci. **b** Quantification of foci associated to DNA Damage in the nucleus of NeuN$^-$ cells. Values represent means ± SEM of the measurements carried out in at least ten fields per sample. Data were analyzed using Kruskal-Wallis

followed by Dunn's post-test; ****p<0.001. White bars with circles represent data from healthy mice, orange bars with triangles represent data from mice with HSV-1 infection, blue bars with diamonds represent data from mice with EAE and purple bars with squares represent data from mice with EAE and HSV-1 infection.

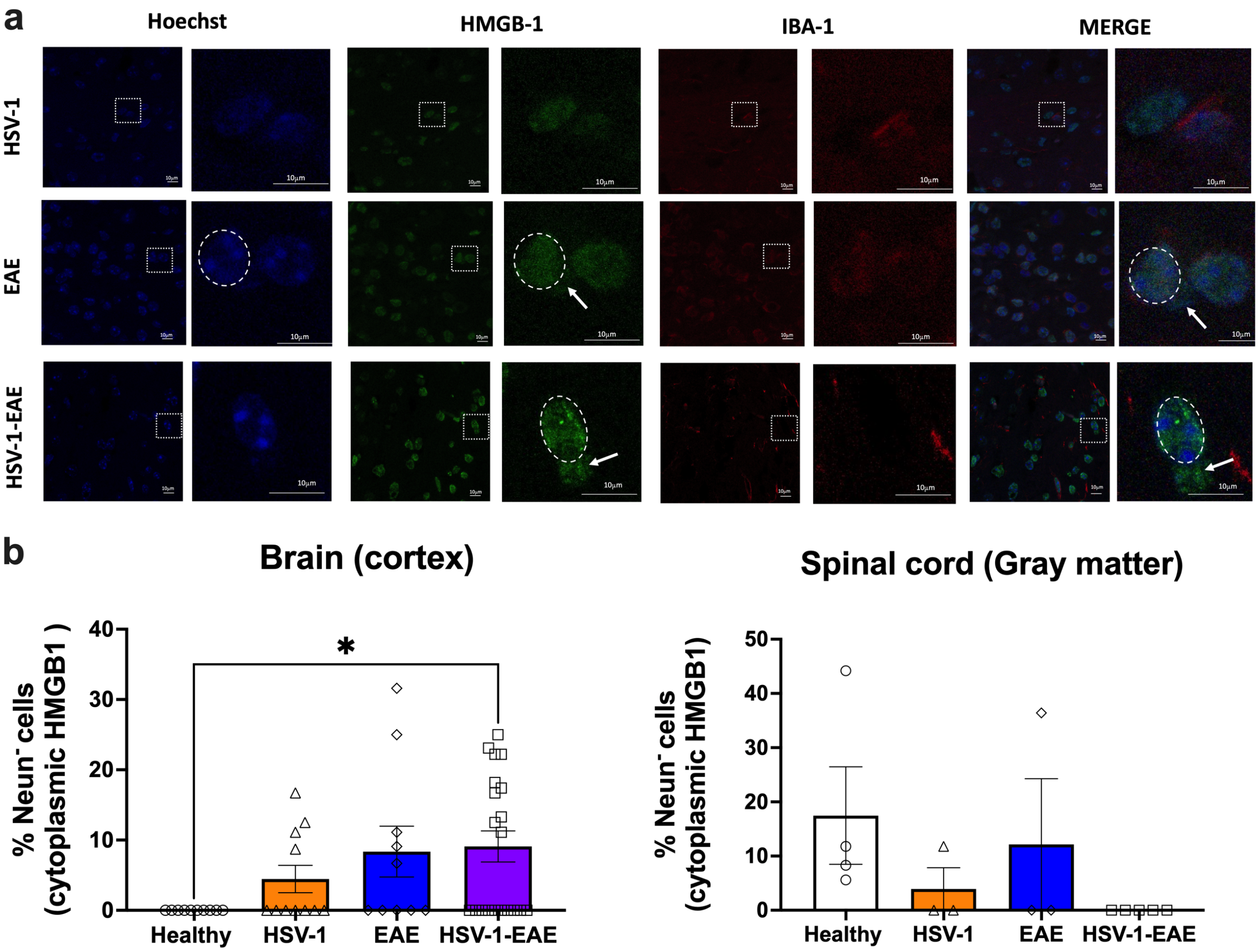


**Supplementary figure 6: The release of HMGB-1 from the nucleus to the cytoplasm is observed in non-neuronal cells in the brains of mice experiencing HSV-1 brain infection and/or EAE.** Brain and spinal cord tissues were harvested 14 days after EAE induction, 45 days after HSV-1 infection, or from mock-treated mice for detecting the expression and release of HMGB-1 by immunofluorescence. **a** Representative images showing Hoechst nuclei staining (blue color), HMGB-

1 staining (green color), IBA-1 staining (red color, an activated microglia marker), and image merges. Left: images for each fluorescence channel correspond to 100X magnifications and right: images are shown at a 5X optic zoom of the area outlined in squares with white dashed lines. Scale bars = 10 μm. White arrows show the translocation of HMGB-1 from the nucleus to the cytoplasm. **b** Graphs show the percentage of NeuN⁻ cells with HMBG-1 release from the nucleus toward cytoplasm in the brain cortex (left) and in the gray matter of the spinal cord (right). Values represent means ± SEM of the measurements carried out in at least ten fields in the brain tissues and three fields in the spinal cord tissues per sample. Data were analyzed using Kruskal-Wallis followed by Dunn's post-test; *p<0.05. White bars with circles represent data from healthy mice, orange bars with triangles represent data from mice with HSV-1 infection, blue bars with diamonds represent data from mice with EAE and purple bars with squares represent data from mice with EAE and HSV-1 infection.

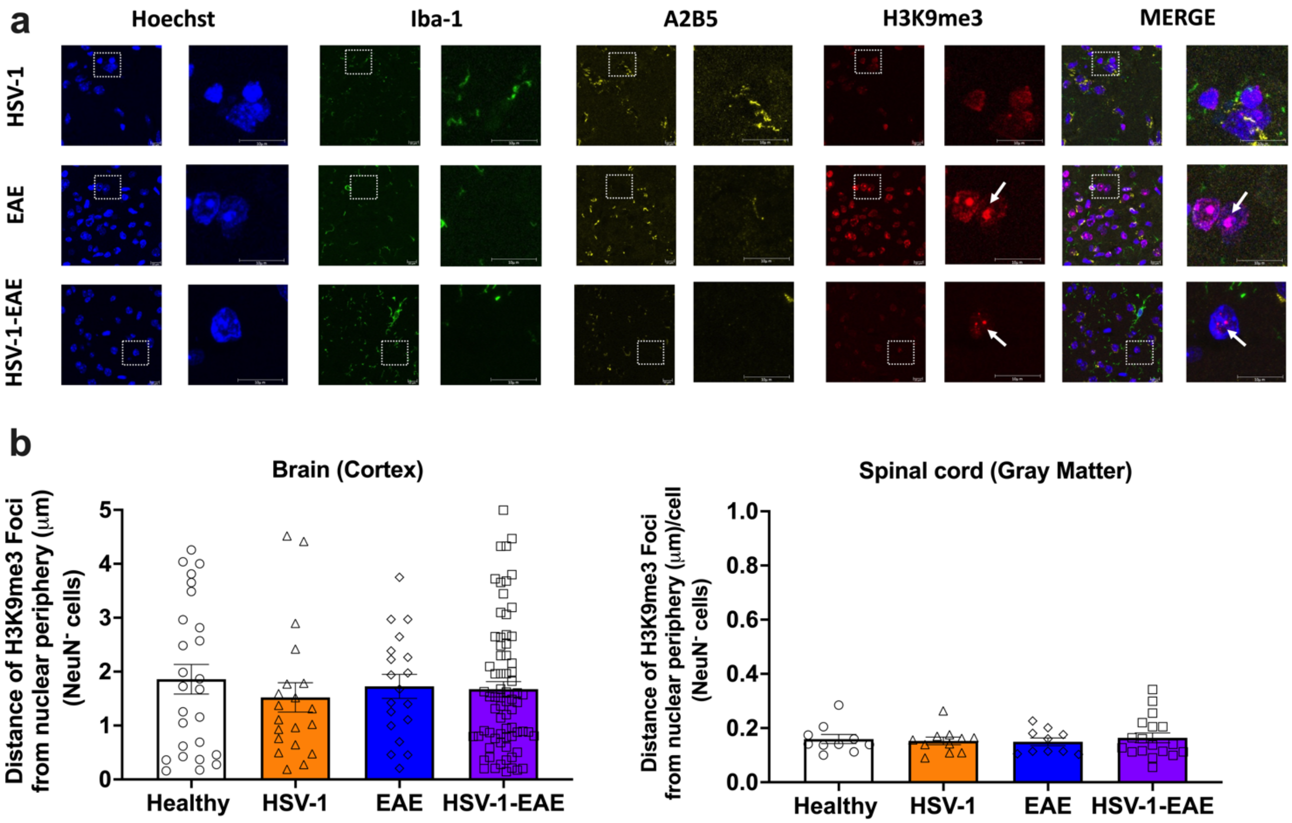

**Supplementary figure 7: H3K9me3-associated foci are sparse in non-neuron cells in the brain and spinal cord tissues of mice with asymptomatic HSV-1 brain infection and/or EAE.** Brain and spinal cord tissues were harvested 14 days after EAE induction, and 45 days after HSV-1 infection, or from mock-treated mice for detecting the expression of H3K9me3 foci by immunofluorescence. **a** Representative images showing Hoechst nuclei staining (blue color), IBA-1 staining (green color, microglia marker), A2B5 staining (yellow color, an oligodendrocyte progenitor marker), H3K9me3 staining (red color), and image merges. Left: images in each fluorescence channel correspond to 100X magnifications, and right: Images are shown at a 5X optic zoom of the area outlined in squares with white dashed lines. Scale bars = 10 $\mu$m. White arrows show senescence-associated heterochromatin foci (SAHF). **b** Quantification of the distance of each H3K9me3 foci from the nuclear periphery in NeuN-negative cells. Values represent means ± SEM of the measurements carried out in at least ten fields in the brain tissues and three fields in the spinal cord tissues per sample. Data were analyzed using Kruskal-Wallis followed by Dunn's post-test. There were no statistically significant differences between groups. White bars with circles represent data from healthy mice, orange bars with triangles represent data from mice with HSV-1 infection, blue bars with diamonds represent data from mice with EAE and purple bars with squares represent data from mice with EAE and HSV-1 infection.
