# Supplementary material for: Asymptomatic herpes simplex virus brain infection elicits cellular senescence phenotypes in the central nervous system of mice suffering multiple sclerosis-like disease
Source: Commun Biol. 2024 Jul 4;7:811. doi: 10.1038/s42003-024-06486-x (PMC11224417; doi:10.1038/s42003-024-06486-x)
Supplement: Supplementary file 4 — Reporting Summary [file 42003_2024_6486_MOESM4_ESM.pdf]

nature portfolio

Corresponding author(s): Dr. Pablo González

Last updated by author(s): May 28, 2024

Reporting Summary

Nature Portfolio wishes to improve the reproducibility of the work that we publish. This form provides structure for consistency and transparency in reporting. For further information on Nature Portfolio policies, see our Editorial Policies and the Editorial Policy Checklist.

Statistics

For all statistical analyses, confirm that the following items are present in the figure legend, table legend, main text, or Methods section.

n/a	Confirmed	
☐	☒	The exact sample size (*n*) for each experimental group/condition, given as a discrete number and unit of measurement
☐	☒	A statement on whether measurements were taken from distinct samples or whether the same sample was measured repeatedly
☐	☒	The statistical test(s) used AND whether they are one- or two-sided<br>*Only common tests should be described solely by name; describe more complex techniques in the Methods section.*
☐	☒	A description of all covariates tested
☐	☒	A description of any assumptions or corrections, such as tests of normality and adjustment for multiple comparisons
☐	☒	A full description of the statistical parameters including central tendency (e.g. means) or other basic estimates (e.g. regression coefficient) AND variation (e.g. standard deviation) or associated estimates of uncertainty (e.g. confidence intervals)
☐	☒	For null hypothesis testing, the test statistic (e.g. *F*, *t*, *r*) with confidence intervals, effect sizes, degrees of freedom and *P* value noted<br>*Give P values as exact values whenever suitable.*
☐	☒	For Bayesian analysis, information on the choice of priors and Markov chain Monte Carlo settings
☐	☒	For hierarchical and complex designs, identification of the appropriate level for tests and full reporting of outcomes
☐	☒	Estimates of effect sizes (e.g. Cohen's *d*, Pearson's *r*), indicating how they were calculated

Our web collection on statistics for biologists contains articles on many of the points above.

Software and code

Policy information about availability of computer code

Data collection	The images were processed using Leica Application Suite X (LAS X) software and analyzed using FIJI-ImageJ software (U.S. National Institutes of Health).
Data analysis	Statistical analyses were conducted using GraphPad Prism Version 9.4.1. (GraphPad Software).

For manuscripts utilizing custom algorithms or software that are central to the research but not yet described in published literature, software must be made available to editors and reviewers. We strongly encourage code deposition in a community repository (e.g. GitHub). See the Nature Portfolio guidelines for submitting code & software for further information.

Data

Policy information about availability of data

All manuscripts must include a data availability statement. This statement should provide the following information, where applicable:

- Accession codes, unique identifiers, or web links for publicly available datasets
- A description of any restrictions on data availability
- For clinical datasets or third party data, please ensure that the statement adheres to our policy

All data generated during this study are included in the published article and supplementary files, further inquiries can be directed to the corresponding author/s.

Human research participants

Policy information about studies involving human research participants and Sex and Gender in Research.

Reporting on sex and gender	This study does not involve human research participants
Population characteristics	This study does not involve human research participants
Recruitment	This study does not involve human research participants
Ethics oversight	This study does not involve human research participants

Note that full information on the approval of the study protocol must also be provided in the manuscript.

Field-specific reporting

Please select the one below that is the best fit for your research. If you are not sure, read the appropriate sections before making your selection.

☒ Life sciences ☐ Behavioural & social sciences ☐ Ecological, evolutionary & environmental sciences

For a reference copy of the document with all sections, see nature.com/documents/nr-reporting-summary-flat.pdf

Life sciences study design

All studies must disclose on these points even when the disclosure is negative.

Sample size	The sample size for each measurement is detailed in the figure legends. The sample size is based on sample availability which was determined using statisticall analyis (animal number calcultaed based on experimental variability observed in similar experiments from the research group and in the literature). Statistical analysis for each result is described and reported.
Data exclusions	The data from the immunofluorescence analysis was imported into GraphPad Prism Version 9.4.1., and the ROUT method was used with a Q cutoff of 1% for eliminating outliers.
Replication	Each individual mouse was considered as the experimental unit and numerous experimental units were used per experiment (detailed in the manuscript). Where indicated, experiments have replicates consisting on independent assays performed with different experimental units. As described in the methods and figure legends, images and measurements were obtained from samples obtained from each mouse.
Randomization	Mice were randomly distributed into the different experimental groups before undergoing infection or EAE.
Blinding	The data analysis of the immunofluorescence assays was performed by an observer blinded to specimen identity as described in the manuscript.

Reporting for specific materials, systems and methods

We require information from authors about some types of materials, experimental systems and methods used in many studies. Here, indicate whether each material, system or method listed is relevant to your study. If you are not sure if a list item applies to your research, read the appropriate section before selecting a response.

Materials & experimental systems

n/a	Involved in the study	
☐	☒	Antibodies
☒	☐	Eukaryotic cell lines
☒	☐	Palaeontology and archaeology
☐	☒	Animals and other organisms
☒	☐	Clinical data
☒	☐	Dual use research of concern

Methods

n/a	Involved in the study	
☒	☐	ChIP-seq
☒	☐	Flow cytometry
☒	☐	MRI-based neuroimaging

Antibodies

Antibodies used	1) anti-γH2A.X (Millipore #05-636); 2) anti-NeuN (Abcam, ab177487); 3) anti-IBA-1 (Cell Signaling #17198); 4) anti-A2B5 (Sigma, MAB312); 5) anti-HMGB1 (R&D system, MAB16901); 6) anti-H3K9m3 (Novusbio # 6F12-H4); 7) anti-53BP1 (Novusbio # 100-304)

Validation	1) We have previously validated the specificity of this antibody in https://doi.org/10.1096/fj.202002253RRR.<br>2) Product website:https://www.abcam.com/en-cl/products/primary-antibodies/neun-antibody-epr12763-neuronal-marker-ab177487. Product citations: 436.<br>3) Product website: https://www.cellsignal.com/products/primary-antibodies/iba1-aif-1-e4o4w-xp-rabbit-mab/17198. Product citations: 112.<br>4) Product website: https://www.sigmaaldrich.com/CL/es/product/mm/MAB312. Product citations: 31.<br>5) Product website: https://www.rndsystems.com/products/human-mouse-rat-hmgb1-hmg-1-antibody-951420_mab16901. Product citations: 12.<br>6) Product website: https://www.novusbio.com/products/histone-h3-antibody-6f12-h4_nbp1-30141. Product citations: 27.<br>7) Product website: https://www.novusbio.com/products/53bp1-antibody_nb100-304. Product citations: 700.

Animals and other research organisms

Policy information about studies involving animals; ARRIVE guidelines recommended for reporting animal research, and Sex and Gender in Research

Laboratory animals	C57BL/6 female mice sourced from The Jackson Laboratory
Wild animals	This study did not involve wild animals.
Reporting on sex	We have opted for using female mice in this study due to previous reports indicating a lower susceptibility to EAE by male mice. Moreover, as discussed in the Discussion section of the manuscript, previous studies using a model of eye HSV-1 infection without scarification have reported the presence of senescence markers at the mRNA level specifically in the brainstem of female mice, as opposed to male mice (Sivasubramanian MK, Monteiro R, Harrison KS, Plakkot B, Subramanian M, Jones C (2022). Herpes Simplex Virus Type 1 Preferentially Enhances Neuro-Inflammation and Senescence in Brainstem of Female Mice. J Virol, 96:e01081-22).
Field-collected samples	None
Ethics oversight	The protocols employed in this study were approved by the Scientific Ethical Committee for Animal and Environmental Care of the Pontificia Universidad Católica de Chile and the institutional Biosafety Committee (Protocols #170705018 and #210425003).

Note that full information on the approval of the study protocol must also be provided in the manuscript.
